# Polychlorinated Biphenyls, Oxidative Stress, and Brain Health: Mechanistic Links to Neurodegenerative and Neurodevelopmental Diseases

**DOI:** 10.3390/antiox15020242

**Published:** 2026-02-12

**Authors:** Aurelio Minuti, Alessia Floramo, Veronica Argento, Ivan Anchesi, Claudia Muscarà, Marco Calabrò, Serena Silvestro

**Affiliations:** 1 IRCCS Centro Neurolesi “Bonino-Pulejo”, Via Provinciale Palermo, Contrada Casazza, 98124 Messina, Italy; 2 Department of Biomedical and Dental Sciences and Morphofunctional Imaging, University of Messina, 98125 Messina, Italy

**Keywords:** polychlorinated biphenyls (PCBs), neurodegenerative diseases, oxidative stress, neurotoxicity, molecular pathways, epigenetic alterations

## Abstract

Polychlorinated biphenyls (PCBs) are persistent organic pollutants that remain widely detectable in the environment and human tissues decades after their ban, raising concerns for brain health. Both dioxin-like (DL) and non-dioxin-like (NDL) congeners interfere with neuronal function through partially distinct pathways, including aryl hydrocarbon receptor activation, disruption of calcium and dopaminergic signaling, oxidative stress, and epigenetic remodeling. Experimental and epidemiological studies indicate that developmental PCB exposure is associated with impaired cognition, attention, motor function, and increased risk of neurodevelopmental disorders. Furthermore, chronic exposure in adulthood has been linked to neurodegenerative diseases. At the cellular level, NDL-PCBs sensitize ryanodine receptors, alter dendritic and axonal growth, promote mitochondrial dysfunction, generate reactive oxygen and nitrogen species, and compromise blood–brain barrier integrity, thereby fostering neuroinflammation, synaptic dysfunction, and neuronal loss. This review synthesizes current evidence on the molecular and cellular mechanismtable s underlying PCB-induced neurotoxicity across the lifespan, highlighting oxidative stress as a central factor, integrating calcium dysregulation, neurotransmitter imbalance, and apoptotic and epigenetic pathways. Finally, potential neuroprotective roles of antioxidant strategies are discussed, emphasizing their relevance for mitigating PCB-related neurodevelopmental and neurodegenerative risk.

## 1. Introduction

Polychlorinated biphenyls (PCBs) are a structurally related class of 209 organochlorine compounds, individually referred to as congeners, that were mass-produced from the late 1920s for various industrial and commercial applications, including use as coolants and insulators in transformers and capacitors, plasticizers in PVC coatings, flame retardants, and hydraulic fluids [1]. Over the twentieth century, PCBs were produced on a massive scale, and a considerable proportion of the original stock is still present in soils, sediments, and biota, contributing to ongoing human exposure [2]. Due to their persistence, bioaccumulation, and demonstrated carcinogenic, endocrine-disrupting, and neurotoxic effects, the commercial production of PCBs was banned in the United States in 1979 and later internationally regulated under the Stockholm Convention on Persistent Organic Pollutants (POPs) in 2001, with subsequent amendments in 2008 and 2014 [3]. Despite these bans, PCBs remain ubiquitous environmental contaminants due to their chemical stability, resistance to biodegradation, and continuous release from old electrical equipment, building materials, and industrial waste sites [4,5,6]. They are still detected in soil, sediments, air, wildlife, and human tissues, reflecting their persistence and bioaccumulative nature [7,8,9]. Exposure to PCBs occurs through multiple routes, including occupational contact, inhalation near contaminated areas, use of legacy products, and consumption of contaminated food [10]. Epidemiological and toxicological studies have identified the brain as a particularly vulnerable target for PCB-induced toxicity [11,12,13,14,15]. Both dioxin-like (DL) and non-dioxin-like (NDL) congeners have been shown to interfere with neuronal function, although they act through distinct mechanisms. Among the multiple mechanisms implicated, oxidative stress and redox imbalance emerge as key drivers linking PCB exposure to neuronal dysfunction and brain vulnerability. The DL-PCBs primarily activate the aryl hydrocarbon receptor (AhR), leading to changes in gene expression, while the NDL-PCBs exert their neurotoxic effects through disruption of dopaminergic neurotransmission, calcium signaling, and oxidative stress pathways [16,17,18,19]. Human and animal studies consistently report that developmental exposure to PCBs is associated with adverse neurobehavioral outcomes, including deficits in learning, memory, attention, and motor coordination [20,21,22]. Prenatal PCB exposure has been linked to reduced birth weight [23], lower intelligence quotient (IQ) [24], and impaired visual memory [25]. Moreover, exposure through breastfeeding has been associated with decreased motor activity in offspring of mothers with elevated PCB levels [26]. In contrast, developmental exposure via drinking water has been associated with hyperactivity in adult mice [27]. Accumulation of PCBs in fetal brain tissue following maternal exposure has also been documented [28]. Experimental models further confirm the neurotoxic potential of PCBs: in rodents, exposure to PCB-52 or PCB-180 interferes with the development and neural signaling of the auditory brainstem [29], while in vitro studies have shown PCB-induced neuronal death [30] and interference with estrogen-mediated neuroprotection [31]. PCB exposure has also been found to affect the VEGF/VEGFR system and cerebrospinal fluid production in ovariectomized sheep [32].

Epidemiological studies have linked PCB exposure to neurodegenerative diseases, though results remain partially inconsistent. Some retrospective and case–control studies found elevated PCB levels in post-mortem brain tissue of Parkinson’s disease (PD) and Alzheimer’s disease (AD) patients, especially among women [33], while others reported no association between serum PCBs and PD risk [34]. Nonetheless, occupational exposure to PCBs has been correlated with increased PD mortality in female workers [35]. Moreover, exposure to PCBs during critical developmental windows has been associated with a higher risk of neurodevelopmental disorders such as autism spectrum disorder (ASD) and attention-deficit/hyperactivity disorder (ADHD) [36], supporting the role of PCBs as environmental risk factors influencing cognitive and behavioral dysfunction.

**Figure 1:**
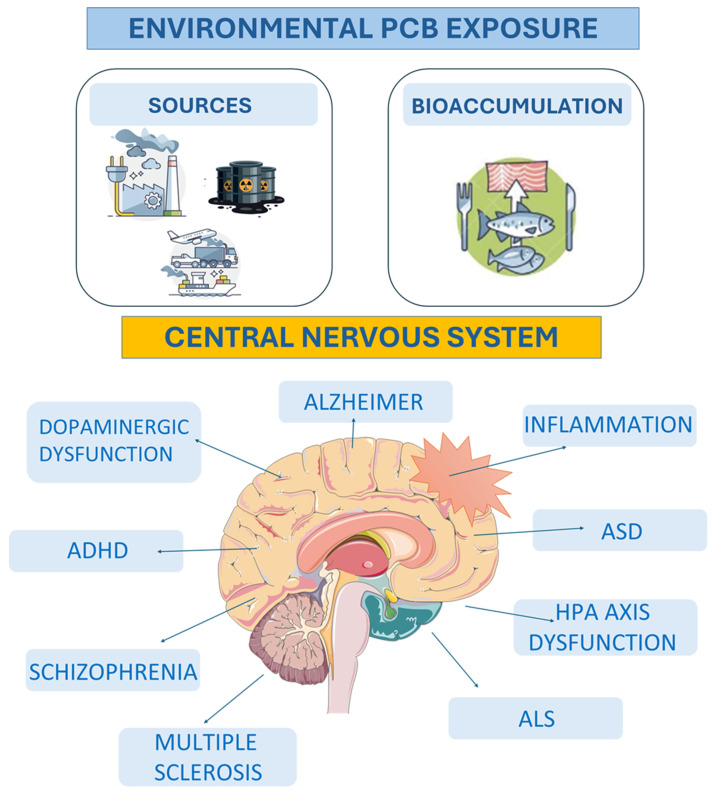
**Main sources of production/exposure to PCBs and their neurotoxic effects on the central nervous system (CNS).** This figure illustrates how environmental exposure to PCBs from various sources and through bioaccumulation in the food chain leads to their accumulation in the CNS, where they are implicated in dopaminergic dysfunction, neuroinflammation, and HPA axis dysregulation, ultimately contributing to a range of neurodevelopmental and neurodegenerative conditions including ADHD, ASD, schizophrenia, MS, ALS, and AD. The image was created using the image bank of Servier Medical Art (Available online: http://smart.servier.com/, accessed on 20 December 2025), licensed under a Creative Commons Attribution 4.0 (CC BY 4.0) (Available online: https://creativecommons.org/licenses/by/4.0/, accessed on 20 December 2025). AD: Alzheimer’s disease; ADHD: Attention-Deficit/Hyperactivity Disorder; ALS: Amyotrophic Lateral Sclerosis; ASD: Autism Spectrum Disorder; CNS: Central Nervous System; HPA: Hypothalamic–Pituitary–Adrenal (axis); MS: Multiple Sclerosis; PCBs: Polychlorinated Biphenyls.

Collectively, epidemiological and experimental evidence identify PCBs as persistent, bioaccumulative neurotoxicants that harm both the developing and adult brain, underscoring their ongoing public health relevance. In the following sections, we will summarize PCB chemistry, classification, and exposure patterns, then discuss how PCBs affect molecular and cellular mechanisms, emphasizing oxidative stress, calcium dysregulation, and dopaminergic dysfunction, and finally integrate human and preclinical evidence linking PCBs to neurodegenerative and neurodevelopmental disorders (Figure 1). Few reviews have systematically integrated molecular mechanisms of PCB-induced oxidative stress with clinical and experimental evidence across both neurodevelopmental and neurodegenerative disorders, a gap this article aims to address.

## 2. Study Selection Methods for the Review

We performed a literature search using PubMed to identify preclinical and clinical studies related to exposure to PCB and neurodegeneration, neurodevelopment, and psychiatric diseases. The following keywords were used in various combinations: “Parkinson”, “Alzheimer”, “multiple sclerosis”, “amyotrophic lateral sclerosis”, “autism”, “schizophrenia”, “anxiety”, “depression” and “PCB” or “polychlorinated biphenyls”. In addition, the reference lists of relevant review articles on PCBs and neurological diseases were screened to ensure that all eligible studies were included. Only articles published in English were considered.

## 3. Chemical Structure and Classification of PCBs

PCBs are a class of synthetic organic chemicals consisting of a complex mixture of chlorinated biphenyl isomers, or congeners, that differ in the number and position of chlorine atoms on the biphenyl rings. Each congener is identified by a specific number from 1 to 209, reflecting an increasing degree of chlorination [37]. Congeners with four or fewer chlorine atoms are defined as low-chlorinated PCBs (LC-PCBs), whereas those containing more than four chlorine substituents are known as high-chlorinated PCBs (HC-PCBs) [38]. Beyond their degree of chlorination, PCBs can undergo transformation in biological systems through hydroxylation or sulfonation, generating hydroxylated (OH-PCBs) and sulfated PCB derivatives, respectively. Notably, 19 congeners are stable atropisomers or enantiomers, exhibiting axial chirality around the biphenyl bond [38]. Each enantiomer and metabolite can interact differently with biological targets, contributing to the diverse toxicological profiles of PCBs and explaining, at least in part, the complexity of PCB-induced neurotoxicity.

Volatility is also structure-dependent: LC-PCBs are more volatile, while HC-PCBs, due to their higher chlorination, are less volatile but exhibit a greater tendency to bioaccumulate and biomagnify along the food chain [38]. Consequently, humans are primarily exposed to HC-PCBs through diet, particularly via fish consumption, and to LC-PCBs mainly through inhalation, especially in urban environments and indoor air [38]. Interestingly, the LC congener PCB 11 has been detected in commercial dairy products from Northern California [39], while the HC congener PCB 95 ranks among the most abundant PCBs in the air of U.S. schools.

Structurally, PCBs are also classified according to the planarity of their biphenyl rings, determined by the presence and position of ortho-chlorine substituents. Congeners lacking ortho-chlorines exhibit a coplanar geometry, allowing efficient binding to the AhR, the canonical receptor for 2,3,7,8-tetrachlorodibenzo-p-dioxin (TCDD) [40,41,42].

**Figure 2:**
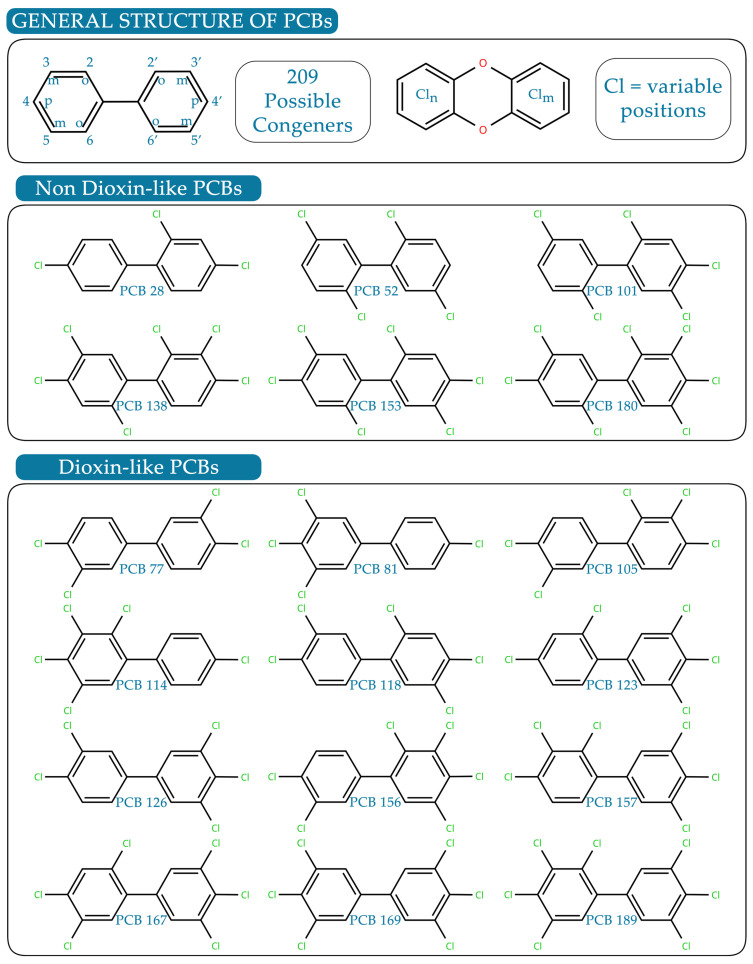
**Basic chemical structure of PCBs and distinction between DL-PCBs and NDL-PCBs congeners.** PCBs share a basic aromatic structure composed of biphenyl, which can be substituted with chlorine atoms at different positions. DL-PCBs have chlorine substitutions mainly at the meta (3, 3′, 5, 5′) and para (4, 4′) positions, with one or no chlorine atoms in the ortho positions (2, 2′, 6, 6′), allowing the biphenyl rings to adopt a coplanar conformation and bind to the AhR. In contrast, NDL-PCBs contain more than one chlorine atom in the ortho positions, causing a torsion of the biphenyl bond and resulting in a non-coplanar conformation with little or no AhR binding affinity. Chemical structures depicted in the figure were generated using the PubChem database (accessed on 20 December 2025). Ahr: aryl hydrocarbon receptor; DL-PCBs: dioxin-like; NDL-PCBs: non-dioxin-like; PCBs: Polychlorinated Biphenyls.

These coplanar congeners are referred to as dioxin-like PCBs (DL-PCBs), and twelve of them (PCB 77, 81, 105, 114, 118, 123, 126, 156, 157, 167, 169, and 189) belong to this class. DL-PCBs act as AhR agonists, regulating the expression of a wide range of dioxin-responsive genes (Figure 2) [43,44]. Although exposure to dioxins and DL-PCBs has been associated with adverse effects in several organs, including liver, skin, and immune system dysfunction [45,46,47,48], limited experimental and clinical evidence supports a direct role of DL-PCBs in neurodevelopmental toxicity. Preclinical studies have shown that TCDD exposure disrupts neuronal migration [49] and ultrasonic vocalizations in mice [50], though at doses exceeding those that cause systemic toxicity. Emerging evidence also links DL compounds to an increased risk of neurodegenerative diseases, notably amyotrophic lateral sclerosis (ALS) [51], characterized by cytoplasmic accumulation of phosphorylated, insoluble Transactive response DNA binding protein 43 kDa (TDP-43) aggregates in neurons. AhR-dependent pathways appear to mediate TDP-43 upregulation in neuronal tissues, yet it remains unclear whether environmental levels of DL-PCBs can trigger similar molecular effects in the human brain [51].

While DL-PCBs mainly act through AhR-dependent pathways, their contribution to neurodevelopmental toxicity appears limited compared with that of NDL-PCBs. In contrast, non-coplanar PCBs, referred to as NDL-PCBs, display little to no affinity for the AhR but are strongly implicated in neurotoxic outcomes [52,53].

Importantly, NDL-PCBs are the predominant congeners detected in human serum, adipose tissue, breast milk, and brain tissue of children diagnosed with neurodevelopmental disorders [52,54]. The widespread detection of NDL-PCBs underscores their persistence, bioaccumulation, and neuroactive potential, positioning them as key contributors to neurodevelopmental and neurodegenerative disorders and as primary targets of the mechanistic discussion in this review.

## 4. Exposure and Persistence of PCBs

Although banned in most countries since the late 1970s, PCBs remain a major environmental concern because of their exceptional chemical stability, lipophilicity, and continued human exposure through diet, especially high-fat foods such as meat and fish, and inhalation of contaminated indoor air. Because PCBs bioaccumulate in adipose tissue, species at the top of the food chain, especially marine mammals and fish, constitute a major source of exposure for humans [55]. Populations with marine-based diets, such as the Inuit of Greenland [56,57], show particularly high plasma PCB levels. Interestingly, these populations also show an increased prevalence of PD compared with the general population [58]. Similar trends have shown that meat consumption has been associated with an increased PD risk [58].

In post-mortem analyses, elevated concentrations of specific PCB congeners have been detected in the caudate nucleus of PD patients [33] and higher PCB levels were linked to substantia nigra depigmentation in women with PD. Parallel animal studies demonstrated that PCB exposure induces a reduction in striatal dopamine (DA) transporter levels, supporting a mechanistic connection between PCB accumulation and dopaminergic neurodegeneration [59]. Furthermore, orally exposed mice accumulated PCBs predominantly in the heart, spleen, and large intestine, suggesting a gastrointestinal route of entry potentially associated with body-first Lewy pathology [60].

In addition to dietary intake, inhalation has emerged as a relevant exposure route, particularly in contaminated buildings and industrial settings. Long-term exposure experiments in ferrets demonstrated that airborne PCBs accumulate preferentially in the olfactory bulb, reaching concentrations four times higher than in other brain regions. These observations suggest that inhaled PCBs may enter directly through olfactory sensory neurons and reach the brain via axonal transport [61].

Once absorbed, PCBs exhibit extraordinary persistence in human tissues due to their resistance to metabolic degradation and their storage in lipid-rich compartments. Metabolic elimination occurs mainly through cytochrome P450-mediated oxidation, producing more water-soluble metabolites that are slowly excreted [62,63]. However, the half-lives of individual PCB congeners vary considerably [64], depending on their degree and position of chlorination, as well as biological and environmental factors such as age, gender, body mass index, and exposure type [64,65,66]. In humans, PCBs’ half-lives range from a few years for lower-chlorinated congeners to several decades for highly chlorinated ones, particularly in individuals with low or intermittent exposure levels [67,68]. Occupationally exposed workers show shorter apparent half-lives, likely due to enzyme induction from high exposure levels, whereas background-exposed populations tend to retain PCBs for longer periods [69,70].

These findings underscore the long-term persistence and bioaccumulation potential of PCBs, ensuring that even decades after their ban, human and wildlife exposure remains ongoing. This chronic presence maintains PCBs as a relevant environmental risk factor for neurodegenerative and neurodevelopmental disorders, particularly those associated with oxidative stress, dopaminergic dysfunction, and Lewy body pathology [71,72,73].

## 5. Mechanistic Insights into PCB-Induced Neurotoxicity

PCBs exert neurotoxic effects through multiple, interconnected pathways, including oxidative stress, disruption of calcium and dopaminergic signaling, thyroid hormone perturbation, AhR activation, apoptosis, dendritic dysmorphogenesis, epigenetic changes, blood–brain barrier dysfunction, and endocrine disruption [74]. Gaining insight into these pathways is essential to elucidate how PCB exposure contributes to neurodevelopmental and neurodegenerative disorders. NDL-PCB congeners, particularly ortho-substituted PCBs, disrupt neuronal calcium homeostasis and dopaminergic neurotransmission, whereas DL-PCB congeners primarily act via the AhR [74]. This section summarizes the main mechanisms underlying PCB neurotoxicity, integrating evidence from in vitro, in vivo, and human studies.

### 5.1. Oxidative Stress as a Central Mechanism of PCB-Induced Neurotoxicity

Oxidative stress is a critical mediator of PCB-induced neuronal injury. NDL-PCBs increase intracellular reactive oxygen species (ROS) and reactive nitrogen species (RNS), overwhelming cellular antioxidant defenses and causing oxidative damage to lipids, proteins, and nucleic acids [75,76,77]. In cultured cerebellar granule neurons, exposure to PCB-4, PCB-153, or Aroclor 1254 elevated ROS levels in a concentration-dependent manner, leading to apoptosis, whereas DL-PCBs had minimal effects [78]. Similar effects were observed in primary hippocampal neurons treated with PCB 47 or Aroclor 1254; co-treatment with antioxidants such as α-tocopherol or ryanodine receptors (RyRs) inhibitors prevented apoptosis, highlighting the interplay between oxidative stress and calcium dysregulation [79].

Mechanistically, ROS production may occur downstream of RyR activation. NDL-PCBs stabilize RyRs in their open conformation, promoting Ca^2+^ release from the endoplasmic reticulum (ER) [52], which increases mitochondrial Ca^2+^ uptake, enhances ROS generation, triggers cytochrome c release, and activates caspases [52]. Conversely, ROS can act upstream by oxidizing hyperreactive cysteine residues on RyRs, increasing channel open probability, and forming a feed-forward loop [80,81,82].

In vivo studies confirm these effects. Maternal dietary exposure to Aroclor 1254 (0.1–1 mg/kg/day) during gestation and lactation increased markers of oxidative stress such as 4-hydroxynonenal and 3-nitrotyrosine in multiple brain regions of offspring [83]. In adult rodents, higher doses of Aroclor 1254 elevated oxidative stress markers in the striatum and cerebellum [84]. Astrocytes also contribute to PCB-induced oxidative stress; in vitro exposure of cortical astrocytes to Aroclor 1254 led to increased ROS, oxidized glutathione, and upregulation of antioxidant genes (*PRDX1*, *GSTA2*), suggesting metabolic adaptation to maintain redox balance [85].

Overall, oxidative stress represents a pivotal mechanism linking PCB exposure to apoptosis, synaptic dysfunction, and both developmental and degenerative neural pathology. These findings highlight oxidative stress as a key therapeutic target and support the rationale for antioxidant-based strategies to mitigate PCB-induced neurotoxicity.

### 5.2. PCB-Induced Disruption of Calcium Signaling and Ryanodine Receptor Function

NDL-PCBs are potent modulators of intracellular calcium signaling, which is essential for neuronal differentiation, dendritic arborization, and synaptic plasticity [86,87,88].

These congeners sensitize RyRs, ER-resident intracellular Ca^2+^ channels, locking them in an open state at picomolar-to-nanomolar concentrations [52,89,90]. This sensitization disrupts Ca^2+^ oscillatory patterns, alters synaptic plasticity, and affects Ca^2+^-dependent transcriptional pathways, including Wnt2 and miR132, which regulate dendritic growth [91,92].

In hippocampal neurons, RyR-active congeners such as PCB 95 induce marked dendritic overgrowth and enhanced synaptic excitability, whereas PCB 170 produces biphasic effects with initial excitation followed by synaptic depression. Co-application of GABA antagonists transforms PCB 170-induced depression into hyperexcitability and epileptiform activity, demonstrating the impact of PCB structure on excitatory/inhibitory balance [93]. Pharmacological RyR inhibition, siRNA knockdown, or knock-in mouse models carrying sensitizing RyR mutations confirm the causal role of RyRs in these effects [91,94,95]. Developmental RyRs sensitization by PCB 95 disrupts auditory cortex tonotopy and impairs learning and memory [93,96]. Emerging evidence also implicates RyR dysfunction in neurodegenerative processes, particularly dopaminergic neuron vulnerability in PD via Ca^2+^ dyshomeostasis and ER stress [97,98].

### 5.3. PCB-Induced Thyroid Hormone Disruption

Thyroid hormones (TH) are critical for brain development. PCB exposure, especially during the perinatal period, frequently reduces serum thyroxine (T4) levels across species, including humans [99,100,101]. Animal studies show that perinatal Aroclor 1254 exposure induces hypothyroxinemia accompanied by sensory and motor deficits, partially rescued by T4 supplementation [100].

However, the relationship between TH disruption and cognitive impairment is inconsistent. Some exposures that lower T4 do not impair learning or memory [102], and some congeners induce behavioral deficits without significant T4 changes. OH-PCBs may act as thyroid hormone receptor (THR) agonists or antagonists depending on congener and dose. Thus, TH perturbation likely acts as a contributing, but not primary, mechanism in PCB-related cognitive deficits.

### 5.4. AhR-Mediated Mechanisms

DL-PCBs activate the AhR, a transcription factor regulating xenobiotic metabolism and neurodevelopment [103,104]. AhR signaling affects neuronal proliferation, migration, and dendritic arborization; AhRr knockout or constitutive activation leads to cognitive deficits and altered neurodevelopmental patterns [105,106].

Genetic variants with high-affinity AhR (AhRb) increase susceptibility to PCB-induced neurotoxicity by inducing CYP1A/CYP1B metabolism, which modulates both DL and NDL congeners [107]. Mice exposed gestationally and lactationally to DL/NDL PCB mixtures displayed cerebellar defects, hyperactivity, and learning impairments, with effects most pronounced in *AhRb/Cyp1a2*^−/−^ mice [107,108]. AhR-mediated pathways therefore act directly and indirectly to enhance PCB neurotoxicity.

### 5.5. PCB-Induced Dopaminergic System Dysfunction

NDL-PCBs disrupt dopaminergic neurotransmission, affecting synthesis, storage, and reuptake of DA. In vitro studies show that Aroclor mixtures reduce intracellular DA levels, primarily via ortho-substituted NDL-PCBs [109,110]. In vivo, Aroclor 1254 reduces DA in the striatum and substantia nigra of rodents and nonhuman primates [111,112].

Molecular mechanisms include inhibition of tyrosine hydroxylase [113], dopamine transporter (DAT) and vesicular monoamine transporter 2 (VMAT2) [17,114], and impaired vesicular DA storage, leading to enhanced cytosolic metabolism and DA depletion [16]. Such disruptions produce Parkinson-like neurochemical profiles and may contribute to long-term neurodegenerative vulnerability [115]. This dopaminergic vulnerability may underlie the epidemiological associations between PCB exposure and increased PD risk.

### 5.6. Additional Mechanisms: Apoptosis, Synaptic Morphogenesis, and Epigenetic Regulation

PCB exposure induces neuronal cell death accompanied by caspase-3 activation and TUNEL positivity, markers commonly associated with apoptosis, although other cell death mechanisms cannot be excluded [79,83]. PCBs alter dendritic and axonal growth through RyRs–mediated Ca^2+^ signaling, which regulates intracellular calcium dynamics, and through activation of CREB (cAMP response element–binding protein) and mTOR (mechanistic target of rapamycin), signaling pathways that govern neuronal gene transcription and structural growth, resulting in increased dendritic branching at low nanomolar concentrations [94,116].

Epigenetic mechanisms further contribute to PCB neurotoxicity. Exposure increases *REST* (RE1 Silencing Transcription Factor) expression, recruits *HDAC3* (Histone Deacetylase 3), and represses synapsin-1 transcription, causing synaptic dysfunction [117,118]. PCBs also impair glutamate uptake by downregulating *GLT-1*, enhancing excitotoxic vulnerability [119].

Oxidative stress additionally disrupts blood–brain barrier integrity, reducing tight junction proteins (occludin, claudin-5, ZO-1) and promoting neuroinflammatory processes, which can be partially rescued by antioxidants such as quercetin [120]. Together with oxidative stress, these pathways converge to impair neuronal survival, connectivity, and plasticity, linking PCB exposure to both neurodevelopmental disorders and neurodegenerative diseases.

### 5.7. Human and Preclinical Evidence

Epidemiological studies link prenatal PCB exposure to lower IQ, attentional deficits, and cognitive impairment [121,122]. Animal models corroborate these findings, showing impaired auditory responses (PCB 52, PCB 180) [123], disrupted adrenal development, and increased central nervous system (CNS) and intestinal barrier permeability [124,125]. Non-planar congeners, acting via RyRs sensitization and dopaminergic disruption, appear particularly potent in inducing synaptic hyperexcitability, excitatory/inhibitory imbalance, and long-term neurobehavioral alterations [86,93].

**Figure 3:**
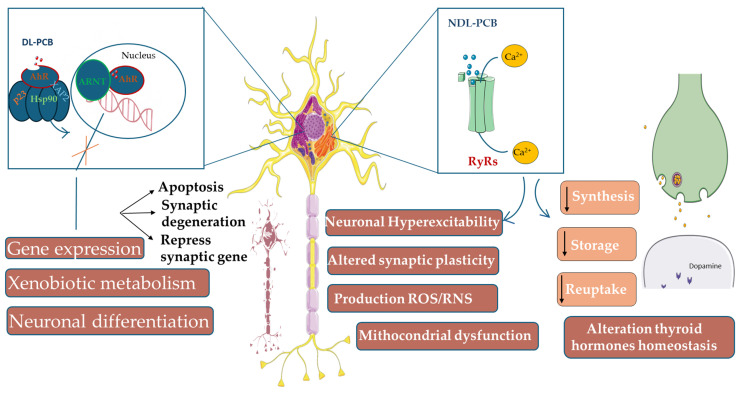
**Schematic representation of the main mechanisms underlying PCB-induced neurotoxicity.** PCBs exert their neurotoxic effects through multiple and interconnected molecular pathways. Non-dioxin-like (NDL) congeners primarily act by sensitizing RyRs, leading to dysregulated intracellular Ca^2+^ release, activation of Ca^2+^-dependent kinases, altered synaptic plasticity, and neuronal hyperexcitability. These events promote excessive production of reactive oxygen and nitrogen species (ROS/RNS), mitochondrial dysfunction, and oxidative stress, resulting in lipid, protein, and DNA damage. Concurrently, PCBs disrupt dopaminergic neurotransmission by reducing dopamine synthesis, storage, and reuptake, and interfere with thyroid hormone homeostasis, which is critical for neurodevelopment. DL PCBs activate AhR, influencing gene expression related to xenobiotic metabolism and neuronal differentiation. Additionally, PCB exposure induces apoptotic pathways, alters dendritic and synaptic morphology, and triggers epigenetic modifications that repress synaptic genes. Collectively, these mechanisms contribute to neuronal dysfunction, impaired cognitive processes, and increased susceptibility to neurodevelopmental and neurodegenerative disorders. The image was created using the image bank of Servier Medical Art (Available online: http://smart.servier.com/, accessed on 20 December 2025), licensed under a Creative Commons Attribution 4.0 (CC BY 4.0) (Available online: https://creativecommons.org/licenses/by/4.0/, accessed on 20 December 2025). DL-PCB: Dioxin-Like Polychlorinated Biphenyl; NDL-PCB: Non-Dioxin-Like Polychlorinated Biphenyl; AhR: Aryl Hydrocarbon Receptor; ARNT: Aryl Hydrocarbon Receptor Nuclear Translocator; Hsp90: Heat Shock Protein 90; RyRs: Ryanodine Receptors; ROS: Reactive Oxygen Species; RNS: Reactive Nitrogen Species.

This integrated view of DL- and NDL-PCB actions on AhR, RyRs, calcium signaling, oxidative stress, thyroid hormones, and dopaminergic neurotransmission highlights how converging molecular pathways ultimately drive synaptic dysfunction and neuronal vulnerability, as depicted in Figure 3.

## 6. PCBs and Neurodegenerative Diseases

Neurodegenerative diseases represent a heterogeneous group of progressive and debilitating disorders of the central and peripheral nervous systems, characterized by selective and irreversible loss of neurons in specific brain regions. Among the most prevalent are AD, PD, and ALS, all of which currently lack effective treatments and impose a substantial social, health, and economic burden. Although the pathogenesis of these diseases remains incompletely understood, it is now well recognized that they result from complex interactions between genetic predisposition and environmental factors, including chronic exposure to neurotoxic and endocrine-disrupting chemicals such as PCBs [126,127].

### 6.1. PCBs Exposure and AD Risk: Experimental Evidence and Epidemiological Limitations

AD is the most common cause of dementia and is neuropathologically defined by amyloid-β plaques, neurofibrillary tangles, and widespread synaptic and neuronal degeneration [128]. Toxicological studies have demonstrated that PCBs exert neurotoxic effects through multiple mechanisms, including oxidative stress, neuroinflammation, mitochondrial dysfunction, and dysregulation of intracellular signaling and neurotransmission, pathways that are also implicated in AD pathogenesis [129].

A particularly relevant mechanism of PCB neurotoxicity involves their interference with hormonal signaling, especially estrogen-mediated neuroprotection. Estrogens, and specifically 17β-estradiol (E2), are known to protect neurons through both genetic and non-genetic mechanisms, including modulation of mitogen-activated protein kinase, Akt, and c-Jun N-terminal kinase (JNK) pathways [130,131,132,133]. Certain PCB congeners, particularly those in the commercial mixture Aroclor 1254, act as endocrine-disrupting chemicals with anti-estrogenic properties capable of attenuating E2-mediated neuroprotection [134,135]. In a preclinical study by Bang et al. [135], the interaction between E2 and Aroclor 1254 was investigated in a cellular model of Aβ-induced neurodegeneration using differentiated cholinergic SN56 neurons exposed to fibrillar Aβ25–35. Treatment with E2 significantly reduced neuronal apoptosis, tau hyperphosphorylation, and JNK activation, events central to AD pathology, through ERα-dependent mechanisms. However, co-exposure to Aroclor 1254 completely abolished these neuroprotective effects, mimicking the action of the selective ERα antagonist ICI182,780. Importantly, Aroclor 1254 does not induce direct cytotoxicity at physiological concentrations, suggesting that it acts as a selective negative modulator of ERα signaling. At the molecular level, Aroclor 1254 impairs E2's ability to suppress tau phosphorylation and JNK activation, key steps in the cascade leading to neuronal dysfunction and death. These findings suggest that PCBs with anti-estrogenic activity may not only exert direct neurotoxic effects but also compromise endogenous neuroprotective mechanisms, thereby increasing neuronal vulnerability to AD-related insults. This is particularly relevant in postmenopausal women, whose reduced endogenous estrogen levels may enhance susceptibility to PCB-induced neurotoxicity [135]. While preclinical data provide compelling mechanistic support for a link between PCB exposure and AD-like pathology, epidemiological studies have yielded inconsistent and often inconclusive results. In the Canadian Study of Health and Aging (CSHA), a population-based investigation including over 2000 adults aged 65 years or older, Medehouenou et al. [136] examined plasma concentrations of several PCB congeners and their association with dementia and AD prevalence. No statistically significant correlations were observed between plasma PCB levels and the presence of dementia or AD, even after adjustment for confounders such as age, sex, education, ApoE4 genotype, lifestyle factors, and comorbidities. Initial inverse associations for PCB congeners 105 and 118 were no longer significant after full adjustment. The authors emphasized that the cross-sectional design limited causal inference and could not assess long-term exposure effects. A subsequent prospective analysis derived from the same CSHA cohort explored the relationship between plasma PCB and organochlorine pesticide (OCPs) levels and cognitive decline over time [137]. Although no direct associations were found between contaminant levels and incident AD or dementia, longitudinal studies using the Modified Mini-Mental State Examination revealed higher plasma concentrations of certain PCB congeners (118, 153, 156, and 163) among individuals who subsequently developed dementia.

These results indicate that, while PCB exposure may not directly predict clinical AD onset, it could contribute to early subclinical cognitive impairment, potentially representing an initial stage in the neurodegenerative continuum. Further complexity arises from occupational cohort studies. The National Institute for Occupational Safety and Health study on more than 24,000 capacitor manufacturing workers in the United States found that cumulative PCB exposure was associated with increased mortality from certain cancers and, in specific subgroups, with neurodegenerative diseases such as ALS, but not with AD mortality [127]. The lack of association with AD may reflect the low incidence of clinically diagnosed cases in the cohort or potential misclassification of cause of death. The findings underscore that PCB-related neurotoxic effects likely depend on exposure duration, congener profile, and individual susceptibility, including genetic and hormonal factors.

Overall, available evidence suggests that PCB exposure, particularly to congeners with anti-estrogenic activity, may contribute to cognitive decline and neurodegenerative vulnerability, potentially influencing AD risk. However, epidemiological evidence remains limited and at times contradictory. It is plausible that PCB-related effects manifest as gradual cognitive deterioration rather than overt clinical AD, influenced by interactions among genetic predisposition (e.g., ApoE4), hormonal status (e.g., estrogen deficiency), and co-exposure to other environmental pollutants. Given the persistence and long biological half-life of PCBs, future longitudinal studies integrating molecular biomarkers, neuroimaging, genetic data, and clinical assessments are crucial to clarify their contribution to AD pathogenesis and cognitive aging [126,127,135,136,137].

### 6.2. PCB Exposure and PD Risk: Experimental, Molecular, and Epidemiological Evidence

PD is the second most common neurodegenerative disorder worldwide, characterized by the progressive loss of dopaminergic neurons in the substantia nigra pars compacta, leading to striatal DA depletion and the emergence of classical motor and non-motor symptoms [138]. While a fraction of PD cases is linked to genetic mutations, most cases are idiopathic, suggesting a substantial role of environmental factors. Among these, PCBs have received growing attention. Epidemiological and experimental studies suggest a link between PCB exposure and PD risk. PCBs can selectively impair dopaminergic neurotransmission by reducing DAT and VMAT2 function, inducing oxidative stress, and downregulating TH, the key enzyme in DA synthesis [59,139,140]. These effects have been demonstrated in animal models and in vitro systems, where PCB exposure alters striatal dopamine metabolism and reduces neuronal density in the substantia nigra, often without causing widespread neurotoxicity [141,142]. Epidemiological evidence indicates that PCB exposure may increase PD risk, particularly in women and in populations with chronic environmental or occupational exposure [33,143].

Preclinical studies provide robust evidence that PCB exposure disrupts nigrostriatal dopamine homeostasis. In a seminal study, male C57BL/6J mice were exposed orally to Aroclor 1254:1260 at 7.5 or 15 mg/kg/day for 3–30 days. While no changes in striatal DA levels or its metabolites, 3,4-Dihydroxyphenylacetic acid and homovanillic acid (HVA) were observed, a dose-dependent reduction in DAT and VMAT2 was detected, with maximal decreases of 35–50% after 14–30 days, preceding neuronal loss [59]. This reduction was specific to the striatum, whereas the substantia nigra and frontal cortex remained unaffected. PCB congeners that accumulated most prominently in the brain included PCB 95, 118, 138, 153, 170, and 180, consistent with their high chlorination and ortho-substitution, which confer persistence and neurotoxicity [111,144,145]. Similar neurochemical and behavioral impairments were observed in adult mice exposed to Aroclor 1254, with increased locomotor activity and selective dopaminergic neuron loss in substantia nigra pars compacta (SNpc) and ventral tegmental area (VTA), alongside oxidative stress and dysregulated iron homeostasis [84].

In vitro studies further elucidate these mechanisms. Organotypic co-cultures of rat ventral mesencephalon and striatum exposed to environmentally relevant PCB mixtures demonstrated early reductions in tissue DA, impaired DAT and VMAT2 function, increased extracellular DA, and progressive loss of dopaminergic (TH-positive) neurons. Notably, GABAergic neurons were affected first, suggesting that disruption of inhibitory support may exacerbate dopaminergic vulnerability [146]. Parallel metabolomic analyses of human populations exposed to PCB 153 indicated alterations in catecholamine metabolism, nitrogen catabolism, mitochondrial function, and oxidative stress pathways, reinforcing the relevance of dopaminergic disruption for PD pathogenesis [147].

Epidemiological evidence linking PCB exposure to PD remains mixed. Case–control studies in the Faroe Islands showed that adult dietary exposure to PCB-contaminated whale meat and fat was associated with increased PD risk, particularly for PCB 101 and other persistent ortho-substituted congeners [148]. Similarly, PCB accumulation in post-mortem brains was higher in PD patients compared with controls, notably for PCB −138, 153, and 180, with sex-specific differences showing stronger associations in women [33,147]. Consistent with this, transcriptomic analyses in peripheral blood from the Enviro-Geno-markers cohort revealed that non-planar PCB congeners (153, 170, 180) altered the expression of PD-relevant genes (e.g., *SNCA*, *PINK1*, *DNAJC6*), particularly in women, suggesting that systemic exposure may induce early molecular signatures of dopaminergic dysfunction [149].

Conversely, prospective studies provide less consistent support. The Finnish Mobile Clinic Health Survey reported an inverse association between serum PCB levels measured decades before PD onset and subsequent risk, potentially reflecting the differential effects of specific congeners, AhR activation, estrogenic activity, or other adaptive mechanisms [34]. Similarly, in a study of 213 participants, serum PCB concentrations were not associated with neurological or neuropsychological performance [150]. Occupational cohort analyses, including >24,000 workers in U.S. capacitor plants, found no overall increase in PD mortality with cumulative PCB exposure, although subgroup analyses suggested potential sex-specific or disease-specific vulnerabilities [127].

Recent molecular studies in human iPSC-derived dopaminergic neurons highlight the biological basis for PCB neurotoxicity. PCB-180 exposure suppressed genes involved in oxidative phosphorylation, synaptic vesicle trafficking, and neuroprotective signaling, and induced mitochondrial dysfunction and oxidative stress, paralleling known mechanisms of dopaminergic neurodegeneration [73]. Complementary studies in primary murine cortical astrocytes demonstrated that low-dose PCB exposure increased oxidative stress and triggered adaptive antioxidant responses, whereas higher doses overwhelmed astrocytic protective capacity, highlighting a potential glial contribution to PD susceptibility [85,151,152].

In summary, converging experimental, molecular, and human data support the concept that PCB exposure—particularly to persistent, ortho-substituted congeners—may disrupt nigrostriatal dopaminergic function, induce oxidative stress, and alter gene expression associated with PD. Epidemiological studies are, however, heterogeneous, reflecting differences in exposure timing, congener composition, genetic susceptibility, and methodological limitations. These findings underscore the need for large, longitudinal studies integrating biomarker-based exposure assessment, molecular endpoints, and sex-specific analyses to clarify the role of PCBs in PD pathogenesis.

### 6.3. PCB Exposure and ALS or Multiple Sclerosis (MS) Diseases: Experimental Evidence and Epidemiological Limitations

Amyotrophic lateral sclerosis (ALS) is a rapidly progressive neurodegenerative disorder marked by motor neuron degeneration, muscle weakness, and eventual paralysis [153]. Several epidemiological studies have indicated an association between PCB exposure and the risk of ALS. For example, a study on 167 ALS patients evaluated plasma concentrations of POPs, including PCB 118, 138, 151, and 175, using an Environmental Risk Score (ERS) to estimate cumulative exposure. Patients in the highest ERS quartile showed a significantly increased risk of mortality, independent of age, sex, and site of symptom onset [154]. Goutman et al. [155] confirmed these findings in a Michigan cohort, showing that individuals with higher cumulative levels of persistent organic pollutants had a markedly greater likelihood of developing ALS and shorter survival, reinforcing the idea that these contaminants exert a substantial combined impact on disease risk and progression.

A central contribution to this field came from Su et al. [156], who investigated the interplay between genetic susceptibility and environmental exposures within the gene–time–environment framework. In this case–control study, conducted in Michigan between 2011 and 2014 and involving 156 ALS patients and 128 controls, the authors evaluated both occupational and environmental exposures to POPs, including OCPs, PCBs, and brominated flame retardants (BFRs), and directly measured blood levels of 122 persistent pollutants. The results showed that cumulative exposure to pesticides strongly increased ALS risk, and that military service was also associated with higher risk across several exposure windows. Importantly, two PCB congeners, PCB 151 and PCB 202, were significantly associated with increased ALS risk, along with three OCPs and two BFRs. Sensitivity analyses in a geographically homogeneous subgroup confirmed the robustness of these associations. The study also highlighted mechanistic plausibility, noting that PCBs can accumulate in the nervous system and disrupt glutamate regulation, calcium homeostasis, and neuronal signaling, ultimately promoting hyperexcitability and apoptosis of motor neurons. Furthermore, authors emphasized the discrepancies between self-reported exposures and measured pollutant concentrations, underscoring the need for biologically grounded exposure assessment. Overall, their findings provide strong evidence supporting PCBs as modifiable risk factors for ALS [156]. These observations are consistent with historical occupational exposure data, in which workers in capacitor manufacturing plants and incidents of PCB-contaminated oil ingestion showed motor conduction abnormalities and increased ALS mortality [127,157]. However, some populations have shown contrasting results. A prospective study in the Faroe Islands, with 28 ALS cases between 1987 and 2009, demonstrated that despite a diet rich in fish and marine products contaminated with PCB and mercury, ALS incidence was not significantly higher than in other European populations. This suggests that exposure alone may not be sufficient to determine risk and that genetic or other environmental factors modulate susceptibility [158]. Similarly, a case–control study conducted in Modena on 38 patients and 38 controls detected PCB in cerebrospinal fluid without robust evidence of associations between continuous contaminant levels and ALS risk, although weak signals emerged in men aged ≥ 60, suggesting possible age-, sex-, or cumulative exposure-dependent effects [159]. Experimental studies indicate that PCBs and other AhR agonists increase TDP-43 levels in both soluble and insoluble fractions and promote the accumulation of proteolytic fragments typical of ALS [51]. Other neurotoxic effects include oxidative stress, mitochondrial dysregulation, and alterations in lipid and xenobiotic metabolism, contributing to neuronal hyperexcitability and motor neuron apoptosis [156,160].

In addition, a prospective study [161] provided one of the first evaluations of PCB exposure in relation to ALS risk using serum samples collected years before disease onset, thereby minimizing the risk of reverse causation typical of retrospective designs. Drawing on three large Finnish cohorts comprising more than fifty thousand individuals and followed for nearly three decades, the study identified incident ALS cases and examined pre-diagnostic serum concentrations of multiple PCB groups. The authors observed a heterogeneous pattern across PCB classes. Notably, higher levels of NDL-PCBs were associated with a reduced risk of ALS, while DL-PCBs showed a non-significant trend toward increased risk, particularly after adjusting for co-exposure to other environmental contaminants. These findings are consistent with mechanistic evidence linking DL-PCBs, via AhR activation, oxidative stress, mitochondrial dysfunction, and increased expression of TDP-43—key pathological features of ALS.

The inverse association was observed for NDL-PCBs; compounds typically regarded as neurotoxic were interpreted as likely confounded by dietary patterns, particularly fish consumption. In Finland during the study period, fish was a primary source of PCB exposure but also a rich source of omega-3 polyunsaturated fatty acids, which possess anti-inflammatory and neuroprotective properties. Thus, the apparent protective association may reflect the beneficial effects of omega-3 fatty acids rather than a direct effect of NDL-PCBs themselves. Overall, the findings of Tang et al. [161] underscore the complexity of PCB exposure in relation to ALS and highlight the importance of disentangling direct toxicant effects from dietary and environmental confounders in future research.

Regarding MS, recent evidence has highlighted a role for OH-PCBs in disease susceptibility. The study by Vaivade et al. [162], identified two specific metabolites, 4-OH-CB187 and 3-OH-CB153, strongly associated with an increased risk of MS. Serum levels of OH-PCBs were significantly higher in patients compared to controls, with more pronounced differences in women with progressive forms. Concerning clinical progression, 3-OH-CB153 showed a borderline association with disability worsening in male MS patients. This suggests a possible modulatory role of PCBs through endocrine and immune effects, consistent with experimental evidence of mitochondrial toxicity, oxidative stress, and aberrant immune response modulation [162,163]. Available evidence indicates that PCBs and OH-PCBs may contribute to both the onset and progression of ALS and MS through multiple mechanisms: oxidative stress, mitochondrial dysfunction, immune alterations, and pathological protein accumulation such as TDP-43. The cumulative effect of exposures to mixtures of contaminants, rather than individual compounds, appears to be a key determinant of risk. Epidemiological and experimental studies highlight the need for integrated approaches, combining direct biological measurements, environmental data, and genetic information to better understand the impact of PCBs on neurodegenerative diseases and to develop effective prevention strategies.

Collectively, the epidemiological, animal, and cellular findings summarized in Table 1, Table 2 and Table 3 provide converging evidence that PCB exposure contributes to PD, ALS, AD, dementia, and related neurodegenerative processes through overlapping dopaminergic, mitochondrial, and oxidative stress pathways.

## 7. PBCs and Mental Disorders

The identification of mental disorders is considerably more intricate, with current psychiatric diagnostics relying largely on the examination of patients’ affective states, behavioral expressions, and cognitive abilities rather than specific biological, genetic or environmental etiological factors [164]. Although imaging methods, neurological examinations, and biochemical or toxicological analyses can complement diagnostic procedures, clear biomarkers for most mental disorders remain absent, except for some dementia cases [165,166,167].

In the next chapters, we will summarize the recent observations of such elements in the context of psychiatric disorders.

### 7.1. PCBs Exposure and Anxiety

A growing number of studies using animal models and human epidemiological data have highlighted how external molecules can influence the brain. As an example, it has been observed that exposure to endocrine-disrupting chemicals, particularly bisphenol A and phthalates, correlate with anxiety-related behaviors [168,169,170,171]. Despite these observations, the range of molecules investigated in this context remains relatively limited, including the data collected for PCBs. Nonetheless, literature data suggests that PCB exposure can influence emotional behavior [172,173]. In this regard, evidence from animal and human studies suggests that PCB exposure can alter emotional behavior by affecting neurotransmitter systems, synaptic plasticity, neuroendocrine signaling, and limbic brain circuits involved in mood and stress regulation. For example, male neonatal mice exposed to PCBs through nursing displayed heightened anxiety-like behavior during both adolescence and adulthood, as assessed by the elevated plus maze and the light-dark box tests, respectively [174]. Adult male Wistar rats exposed to PCB 126 through maternal diet from embryonic day 7 to postnatal day 21 showed increased overall activity in both the elevated plus maze and the light-dark box. In contrast, rats that received a higher prenatal and lactational dose of PCBs (10 mg/kg/day via subcutaneous injection to the dam) did not show any changes in anxiety-related behavior when assessed using a different test, the elevated zero maze [175]. Notably, in Inuit children, exposure to PCB 153 was associated with higher levels of anxiety and feelings of unhappiness and developing anxiety [176,177]. Focusing on the biological mechanisms underlying changes in emotional behaviors, a precise molecular cascade involved with their effect has yet to be identified, mostly due to the intricate neurobiology involved in complex behaviors. Anxiety-like behaviors, for instance, engage multiple neural circuits, many of which are influenced by hormones and exhibit sex-specific differences. Males and females exhibit different reactions to stress [178]. Epidemiological research shows that anxiety and depressive disorders are significantly more prevalent in women than in men, though the factors driving this difference remain unclear [179]. Despite these difficulties, some biological pathways have been suggested as potential bridges connecting PCBs with Anxiety. In this context, the hypothalamic–pituitary–adrenal (HPA) axis, a key regulator of anxiety-related behaviors [180], has been shown to be influenced by PCB exposure; for example, circulating corticosterone levels were reduced in young (postnatal day 15) Sprague-Dawley rats following maternal exposure to Aroclor 1254 throughout gestation. Additionally, PCB exposure was associated with reduced levels of corticotropin-releasing hormone (CRH) and adrenocorticotropic hormone (ACTH), suggesting that the observed decrease in circulating corticosterone levels may originate from hypothalamic alterations [181]; these neuroendocrine changes were accompanied by modifications in social interactions and anxiety-related behaviors during adolescence [182]. Notably, elevated circulating corticosterone levels were observed exclusively in females treated with 0.5 or 1 mg/kg/day of Aroclor 1221, a mixture of PCBs, with no such changes seen in males at either dose level [183]. This implies that the anxiety-related behaviors observed in males may stem from neural mechanisms other than hypothalamic CRH neurons [184]. A study investigated the toxic effects of repeated inhalation of a PCB mixture simulating indoor school air in female rats over 91 days [185]. Exposure resulted in PCB accumulation in multiple tissues (brain, liver, lung, serum, adipose) and was associated with impaired memory, anxiety-like behavior, reduced white blood cell counts, mild plasma metabolomic disruptions, and altered brain gene expression [185]. This study demonstrated that long-term inhalation of school-air PCBs can induce neurobehavioral and systemic toxicity, highlighting potential health risks for children and staff in contaminated school environments.

Hilz et al. (2024) investigated multigenerational anxiety-like behaviors in female rats following prenatal exposure to A1221, a PCB mixture, vinclozolin, or vehicle (Dimethyl sulfoxide) in the F1 generation, with a second exposure administered in the F4 generation [186]. Behavioral effects were absent in the directly exposed F1 offspring but emerged in later generations. Specifically, F4 rats displayed increased anxiety-like behavior, indicating that PCB exposure can induce heritable, lineage-dependent alterations in anxiety-like behavior, and that these effects may be amplified by repeated generational exposures [186].

### 7.2. PCBs Exposure and Major Depressive Disorder

Depression is a common, long-lasting medical condition that impacts a person’s thoughts, emotions, and physical well-being. It is marked by persistent low mood, fatigue, feelings of sadness, sleep disturbances, and a diminished ability to find pleasure in life [187]. Several pieces of evidence link occupational and environmental exposure to PCBs with depression and depressive symptoms [188,189,190]. However, research on potential mechanisms underlying depressive symptoms following PCB exposure is limited.

A key hypothesis for how PCB exposure may lead to depressive symptoms involves disruption of the central DA system [35,191]. DA, along with serotonin and norepinephrine, is a neurotransmitter within the monoaminergic system. These monoamines in the CNS are crucial in the onset and progression of depression. Depressed patients exhibit reduced levels of these neurotransmitters compared to healthy controls [191], suggesting an inverse relationship between DA levels and the severity of depressive symptoms.

Numerous animal studies [142], together with human research, provide evidence that PCBs affect DA signaling. PCBs affect the DA system through multiple mechanisms. A study by Gaum et al. [192] investigates the potential mechanisms linking PCBs exposure to depressive symptoms, focusing on interactions with thyroid hormones and DA metabolism. The authors hypothesized that PCBs may displace T4 from transthyretin, resulting in elevated free T4 (fT4) concentrations. Increased fT4 could influence DA synthesis and metabolism by enhancing tyrosine hydroxylase activity and modulating dopamine turnover, potentially contributing to depressive symptoms. The study analyzed data from 116 participants in the HELPcB cohort, assessing PCB levels, OH-PCBs, fT4 concentrations, HVA excretion, and depressive symptoms across three annual examinations. Cross-sectional analyses revealed significant interactions between lower-chlorinated PCBs, DL PCBs, and HVA levels, with lower chlorinated PCBs showing a longitudinal association. These findings suggest that PCB-induced alterations in thyroid hormone dynamics may mediate the relationship between PCB exposure and depressive symptoms, highlighting the importance of considering thyroid-DA interactions in understanding the neuroendocrine effects of environmental pollutants [192]. A study by Tanner et al. [193] investigated the long-term effects of PCBs on neuropsychological function in older adults from a contaminated area in New York State. The researchers assessed serum PCB levels and neuropsychological function, including verbal memory and depressive symptoms, in 253 individuals aged 55–74 years in 2000–2002. A follow-up assessment was conducted 14 years later with 116 participants. Interestingly, the study showed unexpected (small) improvements in verbal memory and depressive symptoms in subjects with high exposure to PCBs compared to subjects with low exposure, potentially suggesting that the neurotoxic effects of such compounds may not be permanent over time. Nevertheless, the authors have highlighted the possibility of some biases, including unmeasured cofounding factors, such as age, lifestyle, or co-exposure that may affect both PCB levels and depressive symptoms, and healthy survivor selection bias, in which less healthy participants may be underrepresented. Consequently, further longitudinal studies are needed to more accurately assess the long-term neurotoxic effects of PCBs [193].

### 7.3. PCBs Exposure and Schizophrenia

Schizophrenia represents another type of psychiatric disorder, which is characterized by social withdrawal, hallucinations, delusions, and disorganized speech or behavior [194]. Schizophrenia usually emerges in early adulthood, but rarely before the age of 16 years. This emphasizes that, beyond genetic and early development, additional factors occurring later contribute to the development of the disorder [194]. Early neurodevelopment involves the formation of synaptic connections, a process that continues throughout childhood, followed by a shift during adolescence to synaptic pruning, resulting in adults with roughly half the number of synapses of young children [195]. These changes manifest on a larger scale as reduced gray matter volume during adolescence and early adulthood, alongside the simultaneous restructuring of both functional brain networks [196]. A long-standing theory suggests that these developmental processes are altered in schizophrenia, resulting in broad disruptions in neural communication and the emergence of cognitive deficits in affected individuals [197]. Consistent with this view, neuroimaging studies indicated that typical developmental pathways are altered in schizophrenia, marked by greater gray matter reduction and abnormal network organization at the onset of the disorder, which is linked to cognitive impairments [198,199]. Lesiak et al. [91] explored how exposure to the ND-PCB congener PCB 95 affects neuronal connectivity and synapse formation. They used in vitro neuronal cultures, particularly hippocampi of postnatal day 1–2 Sprague-Dawley rats, to show that low concentrations of PCB 95 stimulate dendritic growth and increase dendritic spine density. Notably, they found that PCB 95 elevates expression of miR-132, a microRNA known to promote synaptogenesis, and that this process is mediated via activation of RyRs [91]. Inhibiting RyRs blocked the PCB-induced increases in miR-132 and synaptic growth, suggesting the receptor is a necessary component of the pathway. The authors showed that PCB exposure can perturb neural circuit development by artificially enhancing synaptic connectivity, which over time may lead to maladaptive network organization. Their findings provide a plausible mechanistic link between PCB exposure and alterations in brain connectivity relevant to neurodevelopmental disorders [91].

Conversely, a more recent study by Cheslack-Postava et al. [200] examined whether maternal prenatal exposure to PCBs and the pesticide metabolite DDE is associated with increased risk of schizophrenia in offspring. Using a Finnish birth-cohort design (FIPS-S cohort), the authors identified 500 case–control pairs, each case having at least two diagnoses of schizophrenia or schizoaffective disorder, and matched controls by sex, date of birth, and residence [200]. Maternal prenatal serum samples were assayed for multiple PCB congeners and DDE, and total PCB burden was computed as the sum of congeners. The analyses did not find statistically significant associations between higher maternal PCB or DDE levels and offspring schizophrenia risk.

In conclusion, current evidence provides very limited support for a potential role of PCBs in schizophrenia. While an early cell culture study suggested a possible effect, this finding has not been replicated, and the available human data do not indicate an association. Given the scarcity of research in this area, further studies are needed before any definitive conclusions can be drawn regarding the impact of PCBs on the pathogenesis of schizophrenia.

### 7.4. PCBs Exposure and Bipolar Disorders

Bipolar disorder is a complex, recurrent mood disorder affecting about 2% of the global population, with significant individual and societal impact. Recent research has explored its prevalence, genetic and neurobiological underpinnings, but the connection between PCBs and Bipolar disorder is not directly addressed in the current literature. This disorder is associated with increased rates of inflammatory and autoimmune conditions. Patients often show elevated levels of proinflammatory markers (e.g., TNF-α, CRP, IL-1β), and inflammation is thought to play a central role in BD pathophysiology [201]. Purinergic signaling and HPA axis dysregulation are highlighted as key mechanisms. Interestingly, this axis has been proven to be influenced by PCBs. Nevertheless, no direct studies have investigated the significance of this association in the context of bipolar disorder. These experimental and clinical findings on PCB-related anxiety, depression, and psychosis-like outcomes are summarized in Table 4.

## 8. PCB and Neurodevelopmental Disorders

Neurodevelopmental disorders currently encompass conditions such as intellectual disability, communication disorder, ASD, ADHD, specific learning disorders (SLD), and movement disorders [204]. Diagnosis is often challenging due to significant symptom overlap between different disorders and frequent atypical presentations, making clinical management complex from the outset.

The rising prevalence of neurodevelopmental disorders is a topic of significant interest. While the reason for this trend remains under discussion, the apparent increase is largely attributable to improved symptom recognition and advances in diagnostic methods. In the USA, data from the National Center for Education Statistics show that during the 2015–2016 school year, 6.7 million students, representing 13% of all public-school enrollments, received special education services. Of these, the majority were identified as having SLD, which accounted for 21.5% of all disabilities in 1976–1977 and rose to 34.8% by 2014–2015, maintaining a relatively steady pattern since the 1980s. The prevalence of ADHD is believed to have tripled in recent years. In Italy, the 2003 Consensus Conference held in Cagliari marked the launch of the National Registry and pharmacovigilance initiatives, which officially began in 2007. Estimates suggest that, when considering all degrees of severity, ADHD affects between 0.4 and 3.6% of the population [205,206]. Neurodevelopmental disorders, even when confined to specific learning domains such as ASD, pose a significant health, social, and economic burden due to their high prevalence and complex management [204]. When no clear organic cause can be identified, diagnosis relies on symptom clusters, often leading to diagnostic and etiological challenges. This complexity is often exacerbated by the structure of healthcare systems in industrialized nations, which were originally designed to address acute rather than chronic or complex health issue [207]. In recent decades, particular attention has been paid to environmental exposures as potential modulators for these disorders. Among these, POPs, and in particular PCBs, have emerged as prime candidates capable of altering brain development through multiple molecular and cellular mechanisms. Neuroimmune signaling functions during development can be disrupted not only by inflammatory challenges but also by environmental factors such as maternal stress, a high-fat diet, or environmental pollutants, which influence microglial activity [208,209].

### 8.1. Exposure and Mechanistic Effects of PCBs on Brain Development

PCBs can disrupt neurodevelopmental processes, including neuroimmune signaling, during critical periods of brain maturation. Several epidemiological studies and systematic reviews have examined the link between perinatal exposure to PCBs and their OH-PCBs and neurocognitive deficits in children. The research highlights negative effects on memory, attention, and cognitive development in school-age children, although the evidence on motor development in early childhood is less robust [210]. As previously discussed, PCBs persist in the food chain due to industrial contamination; thus exposure is significant during early development. After ingestion, PCBs are partially metabolized in the liver and converted to OH-PCBs, which are more water-soluble. These metabolites can cross the placenta more easily than the original compounds [211] and can act as potent endocrine disruptors [212]. Notably, the gut microbiota plays an active role in this metabolic process. Recent studies have shown that human gut bacterial species, such as Clostridium, can metabolize PCBs with a specificity that depends on their chemical structure. This mechanism highlights how the complex interaction between environmental contaminants and individual health status (including microbiota balance) can modulate the actual toxicity and availability of neurotoxic metabolites during development. For example, one study compared microbial metabolism with that of human liver cells (HepG2 line), demonstrating that the intestine must also be considered a metabolically active organ for PCBs [213]. Moreover, it is known that PCBs can alter TH signaling in the fetal brain, compromising neurological development through gene expression modulation [214].

PCBs and their OH-PCB metabolites have chemical structures similar to THs, such as T4 and triiodothyronine (T3). Several in vivo experiments have shown that these compounds can therefore affect TH functions and cause brain damage [215]. For instance, placental exposure to 4-OH-2,3,3′,4′,5-pentachlorobiphenyl reduced plasma TH levels in offspring and impaired behavioral development [216]. Unmetabolized PCBs are sequestered in maternal adipose tissue and are mobilized during periods of tissue turnover, such as pregnancy and lactation [38,217]. They cross the placental barrier, potentially affecting fetal development [218]. Recent cohort studies have evaluated prenatal exposure to complex mixtures of environmental pollutants, including PCBs, demonstrating altered levels of maternal and fetal biomarkers such as plasma concentrations of PCBs, TH, and metabolic proteins. Exposure to these mixtures of pollutants during mid-pregnancy is correlated to decreased vitamin D concentrations in the mother, with PM10 and several other atmospheric contaminants showing particularly strong negative associations. These data underscore the importance of considering both the cumulative and individual effects of pollutants on the metabolic and endocrine health of the mother [219]. The prenatal period is crucial for proper brain development, during which broad cognitive, motor, and behavioral aspects are formed [220]. The fetal brain is extremely vulnerable to toxic environmental exposures because its development is time-sensitive, the blood–brain barrier is immature, and compensatory and homeostatic mechanisms are incomplete during gestation [38]. All of this must happen in specific time windows, so if a developmental process in the brain is interrupted or inhibited, the chances of subsequent repair are slim, and the consequences can therefore be permanent [221]. The placenta offers some protection against exposure to chemicals but does not provide effective protection against environmental pollutants [222].

Several studies have explored the link between PCBs and ASD, finding in some cases an association between higher levels and an increased risk of ASD diagnosis or autistic traits. It is hypothesized that PCBs may damage the brain regions responsible for regulating attention and impulse control. The association is often more pronounced in males [223].

Although there is a wealth of literature on this topic, the possibility of reaching definitive conclusions is limited by significant methodological heterogeneity in markers and assessment tools. Nevertheless, the behavioral neurotoxicity of PCBs in development is confirmed in numerous animal and human models [74,224]. These associations are supported by modern longitudinal studies. For example, a cohort of 18-month-old Japanese infants found a significant association between PCB levels in umbilical cord blood (as a marker of prenatal exposure) and an increased risk of ASD assessed at 18 months of age. Furthermore, using a machine learning study that combined PCB levels with information on the infant’s spontaneous body movement, researchers demonstrated that prenatal exposure to PCBs may be associated with the subsequent onset of autistic behaviors [225]. In line with the theory of increased fetal susceptibility, recent studies have also demonstrated a direct impact on fetal anatomy. Higher concentrations of PCBs in maternal serum and umbilical cord blood have been associated with reduced birth weight and head circumference, which is a key indicator of brain development [226,227]. These data establish a clear relationship between prenatal exposure to PCBs and altered neural development before birth, resulting in neurobehavioral deficits. These findings suggest that environmental contaminants such as PCBs contribute to the increased prevalence of neurodevelopmental disorders by disrupting critical developmental signaling pathways and interacting with genetic vulnerability factors [228].

### 8.2. PCB Exposure and ASD: Epidemiological Evidence, Experimental Models, Methodological Limitations/Challenges

ASD is a significant neurodevelopmental condition characterized primarily by ongoing difficulties in social communication and interaction, along with restricted interests and repetitive patterns of behavior. Although there is currently no definitive cure for ASD, research indicates that early intervention in children at risk can significantly improve their social adaptation [229,230]. While ASD has traditionally been viewed as a genetically based disorder [231], research focusing on specific candidate genes has yielded limited results. Consequently, it is now recognized that both genetic and environmental factors, as well as their interactions, must be taken into account when exploring the causes of ASD [232]. ASD has shown an increasing prevalence worldwide. Although the rise may partially reflect better diagnostic awareness and broader criteria, environmental factors likely contribute alongside genetic susceptibility [233]. Moreover, environmental chemicals including PCBs have been linked to immune disturbances that may support the development of ASD in genetically vulnerable fetuses [234]. The CHARGE (Childhood Autism Risks from Genetics and the Environment) study further underscores how genetic background interacts with specific environmental exposures, including PCBs, to shape ASD outcomes [74,235]. The body of evidence linking prenatal chemical exposure to ASD risk continues to grow. A recent meta-analysis, which included 12 studies (totaling 4946 participants), estimated an associated risk (Odds Ratio) of 1.80 for maternal PCB exposure and 1.26 for pesticide exposure during pregnancy and the risk of ASD in children [236]. Similarly, a systematic review confirmed a strong association between early exposure to agricultural pesticides (mainly organochlorines, carbamates, and pyrethroids) and autism, noting that maternal biomarker levels of DDE were higher in ASD cases. However, evidence regarding PCBs was inconsistent, and no clear conclusions could be drawn [237]. However, the picture is complex, particularly when considering chemical mixtures. A case–control study of Jamaican children examined serum concentrations of PCBs and organochlorine pesticides in relation to ASD. Children with ASD had lower serum concentrations of PCB 153 and PCB 180 than typically developing controls [238]. Additionally, the HUMIS (Norwegian Human Milk Study), a prospective birth cohort, analyzed exposure to multiple POPs in breast milk and subsequent ASD diagnosis in children (20 children out of 1199). This study, in contrast to the findings of the Early Markers of Autism (EMA) study, found no positive associations between maternal serum levels of PCBs, DDE, and trans-nonachlor and the risk of ASD in multi-pollutant analyses [239]. Conversely, analyses carried out as part of the EMA study found an increased risk of ASD associated with prenatal exposure to PCB 138/158 [240] and PCB 153 [241]. This divergence suggests that differences in methodology, population, and the approach used for analyzing chemical mixtures significantly influence results [242]. Further complexities emerge from cohort studies analyzing chemical mixtures. An analysis conducted on the Early Autism Risk Longitudinal Investigation (EARLI) cohort, investigating the effect of a mixture of pollutants, including 11 PCBs, on neurodevelopmental scores identified a potentially inverse linear relationship between POP mixtures and Social Responsiveness Scale (SRS) scores, suggesting fewer social deficits with higher quantiles of the mixture. Furthermore, a positive linear relationship was found between POPs mixtures and Mullen Scales of Early Learning—Early Learning Composite (MSEL-ELC) and Vineland Adaptive Behavior Scales (VABS) scores, indicating improved cognitive and adaptive functioning with higher mixture quantiles [242]. The conflicting findings, particularly the protective association observed in the EARLI cohort, underscore the need for advanced statistical models (like BKRM) and larger, more homogeneous cohorts to reliably assess the toxicological effects of complex environmental mixtures [243]. Finally, while PCBs are a major focus, other POPs also contribute to neurotoxicity. In a Vietnamese cohort, perinatal exposure to dioxins (measured in breast milk) was associated with cognitive and motor developmental deficits in children, including lower Bayley-III scores at 3 years of age and alterations in gaze behavior [244]. This highlights that multiple persistent contaminants act through similar neurotoxic pathways.

Limitations of the current studies are that they do not assess the effects of specific types or categories of PCBs, mainly because most studies report only total PCB levels or a small set of common indicators of PCBs. The importance of this limitation is highlighted by a recent study indicating that NDL-PCBs that affect RyRs, rather than DL-PCBs or total PCB levels, show a slight positive association with ASD [245]. Genetic factors probably play a role in how PCBs affect outcomes related to ASD, as shown by a pilot study that found a potential positive link between PCB 153 and ASD in people with a deletion mutation in the gene for the glutathione transferase, but not in those without this mutation [238]. Evidence suggests that individual susceptibility plays a key role. Gene-environment interaction studies show that PCBs amplify neurodevelopmental effects in genetically vulnerable individuals. This idea has been reinforced by Sharifi et al., (2024), who demonstrated in mouse models that PCB exposure can share and amplify the same genetic pathways deregulated by key neurodevelopmental mutations [246].

A recent systematic review and meta-analysis of cohort studies confirmed the existence of positive associations between exposure to PCB 138 and incidence in the development of ASD [247]. A 2020 study found a link between PCB levels in maternal plasma during pregnancy and a higher occurrence of autistic behaviors in children aged 3 to 4 years, based on analyses using Bayesian predictive odds ratios [248].

Additionally, the ability to draw definitive conclusions on POPs is further limited by significant methodological heterogeneity in markers and assessment tools, and by the analytical complexity required to accurately measure the exposome. To address this measurement challenge, one study developed a two-phase analytical method (semi-quantitative screening of 156 substances followed by quantitative targeted analysis of 69) using Triple Quadrupole Gas Chromatography-Mass Spectrometry (GC-MS/MS) on 183 plasma samples, including children with ASD, their parents, and neurotypical controls. This approach allowed for the frequent detection of known persistent pollutants like DDE and PCB congeners (specifically PCB 118 and 180), as well as substances less frequently studied in human plasma, such as isosafrole and hexachlorobutadiene. The capacity to detect a broad spectrum of contaminants provides more robust data for mixture analysis [249]. Another advanced analysis, focusing on epigenetics, utilized data from the Markers of Autism Risk in Babies—Learning Early Signs cohort. This study measured 209 PCB congeners in maternal serum and simultaneously analyzed placental DNA methylation networks (using Weighted Gene Correlation Network Analysis, WGCNA) [250]. The most frequently detected PCB congeners (in ≥50% of samples) in maternal serum were PCB 153 + 168, PCB 170, PCB 180 + 193, and PCB 187. This sophisticated analysis revealed that maternal serum PCB levels were correlated with specific placental DNA methylation modules, and crucially, some of these modules were linked to subsequent childhood neurodevelopmental traits. This highlights that PCBs not only act as direct toxins but can also modulate epigenetic mechanisms that influence neuronal development [250].

Despite these analytical advances, the possibility of reaching definitive, consistent conclusions is still limited. A recent systematic review conducted by Cunha et al. [251] examined 27 observational studies evaluating prenatal exposure to various contaminants, eight of which examined the correlation between PCBs and autistic traits. Overall, no strong correlation emerged between prenatal exposure to these endocrine disruptors and the subsequent onset of autistic traits. These findings contrast with the growing body of evidence from preclinical studies linking early exposure to endocrine disruptors with alterations in neurological development, including autistic-like behaviors. These discrepancies may be due to methodological differences between human studies and the inherent limitations of human studies in addressing the risks of exposure to endocrine disruptors. This highlights the urgent need to standardize exposure measurement and data presentation to fully understand the risk to public health. In this context, it would be useful to supplement prenatal care with the assessment of exposure to endocrine disruptors using tools such as questionnaires. This approach could have a positive impact at the individual and population levels, providing useful information for characterizing and preventing exposure to endocrine disruptors [251].

Experimental studies (including animal models and in vitro cell models derived from human cells) are crucial for shifting from epidemiological association to understanding the mechanisms of action and developmental neurotoxicity of individual substances or mixtures.

An innovative approach proposed for the risk assessment of chemical mixtures suggests the use of mixed neuronal/glial cultures derived from human induced pluripotent stem cells [252]. The study grouped chemicals into mixtures with similar mechanisms of action (e.g., lead (II) chloride, chlorpyrifos, bisphenol A) and dissimilar mechanisms of action (e.g., methylmercury, valproic acid, PCB138). The results confirmed that individual chemicals at non-cytotoxic or very low cytotoxic concentrations can exhibit neurotoxic effects in mixtures. The protein changes, altered synaptogenesis, and reduced neurite length and branching observed in exposed cultures suggest that this approach is a reliable strategy for identifying chemical mixtures with potential developmental neurotoxicity [253].

Furthermore, mechanistic studies are actively exploring the epigenetic basis of PCB toxicity. For instance, an in vivo study in mice showed that prenatal exposure to a human-relevant PCB mixture alters the DNA methylome in both the placenta and fetal brain. Thousands of differentially methylated regions were distinguished from the fetal brain and placenta of PCB-exposed mice from controls. Specifically, the methylated regions were enriched for genes involved in the Wnt and Slit/Robo signaling pathways, which are fundamental to neurodevelopment [254]. Beyond methylation at specific sites, a relatively unexplored area of research is the relationship between PCBs and neurotoxicity through broader epigenetic alterations, such as hypomethylation of repetitive DNA (e.g., Alu and LINE-1 sequences), observed in both the Greenland Inuit population and rat models. Such alterations, together with Copy Number Variations that frequently occur in neurodevelopmental disorders, suggest that PCBs may influence genomic instability, a poorly understood but crucial aspect of etiology [255]. This molecular evidence complements earlier preclinical research in non-human primates and rodents showing that developmental exposure to PCBs impairs cognitive function without causing negative effects on reproduction or birth outcomes [13,15,256,257]. The review concluded that developmental neurotoxicity linked to exposure from legacy PCB mixtures is mainly caused by NDL congeners [2,52].

In short, five studies, including one conducted in mice [258], and four in rats [182,183,259,260], have been published examining how PCBs affect different measures of social behavior. In contrast, the four rat studies showed that perinatal exposure to a mixture of PCBs 47 and 77 [259], or Aroclor 1221 [182,183,260] typically reduced measures of sociability in males and produced varied effects in females, depending on the dosage and timing of exposure [2]. These findings align with the sex differences seen in ASD, but more detailed Structure-Activity Relationship research is needed to better understand how developmental PCB exposures affect neurobehavioral outcomes.

An important unresolved question in the field is how xenobiotic metabolism influences neurotoxic effects that result from developmental exposure to PCBs. Cytochrome P45O enzymes are central to PCB metabolism, transforming the parent compounds into hydroxylated PCBs, which can then undergo further conversion into glucuronide, sulfate, and other conjugated metabolites [38]. Among specific non-dioxin-like congeners, PCB95 has received considerable attention in experimental models. Perinatal exposure to PCB95 stimulates excessive dendritic growth in developing neurons through RyR-mediated calcium signaling [92]. This suggests that PCBs are environmental risk factors for neurodevelopmental disorders, as defects in neuronal connectivity are a recurring pathological hallmark in most of these conditions [261]. Here is experimental evidence showing that exposure to PCB-95 increases certain aspects of ASD, including altered development of the auditory cortex in weaned rats, resulting in an excitation–inhibition imbalance [262] and altered social behavior in rats [259].

Furthermore, the risk is modulated by maternal nutritional status: it has been observed that the association between gestational PCB exposure and autistic behaviors is stronger in women with low folic acid supplementation, suggesting that this micronutrient, essential for methylation and the reduction in oxidative stress, may mitigate the neurotoxic effects of PCBs [263]. Interestingly, using single-nucleus RNA-seq in mosaic female cortex, it was shown that MeCP2 mutation and PCB exposure converge on shared transcriptional pathways and non-cell-autonomous regulatory mechanisms [264].

Together, these epidemiological and experimental findings emphasize that specific PCB congeners and complex contaminant mixtures can influence neurodevelopmental trajectories and ASD-related outcomes through multiple, partially converging pathways, as summarized in Table 5 and Table 6.

### 8.3. PCB Exposure and ADHD-like Neurodevelopmental Outcomes

Among neurodevelopmental disorders, ADHD has been repeatedly associated with prenatal and postnatal exposure to PCB mixtures, with a particular involvement of highly chlorinated NDL congeners. Birth cohort studies have shown that higher PCB levels in maternal blood, cord serum, or breast milk are associated with an increased risk of hyperactive–impulsive symptoms and attentional deficits in children, as demonstrated in the New Bedford cohorts [265] and in the Duisburg birth cohort [266]. In New Bedford, PBPK-based reconstruction of early postnatal PCB-153 exposure suggests that cord serum PCB-153 at birth shows a clearer association with ADHD-related behavioural scores than modelled postnatal exposure, supporting a potentially stronger impact of prenatal exposure [267]. Cross-sectional studies in chronically exposed adolescent populations, such as the Mohawk sample [268], further suggest complex associations between body burden of PCBs, breastfeeding history, and ADHD-related symptom profiles.

Beyond clinical evidence in humans, preclinical data indicate that perinatal or postnatal PCB exposure induces behavioural phenotypes compatible with ADHD. *n* rodents exposed during development to commercial PCB mixtures (e.g., Aroclor 1254) or defined congeners (e.g., PCB-153), studies have reported motor hyperactivity, increased spontaneous activity, altered learning, and executive dysfunction on [269,270]. Such effects have also been observed in genetically vulnerable lines such as Spontaneously Hypertensive Rats (SHR), a widely used animal model of ADHD, supporting gene–environment interactions in PCB-associated neurobehavioural outcomes [270]. Additional work reports changes in activity patterns and social behaviour following perinatal exposure to PCB mixtures, suggesting disruption of neurobehavioural domains overlapping with ADHD-related constructs [11]. This body of work includes studies describing increased locomotor activity and higher operant response rates after postnatal PCB-153 exposure, as well as changes in activity and social behaviour following perinatal exposure to PCB mixtures [271,272].

From a mechanistic perspective, ADHD-related vulnerability appears to arise from the convergence of multiple pathways: sensitization of ryanodine receptors and consequent disruption of calcium-dependent signalling interferes with the maturation of cortical and striatal circuits that govern attention and behavioural inhibition, while dopaminergic dysfunction and oxidative stress may contribute to altering the excitation/inhibition balance in fronto–striato–cerebellar networks, thereby promoting an ADHD-like behavioural phenotype. In vitro and in vivo studies on neurons and brain tissue have shown that NDL congeners such as PCB-95 and PCB-153 modulate ryanodine receptors, disrupt calcium homeostasis, and activate calcium-dependent, Wnt2/miR-132, and mTOR signalling pathways, promoting aberrant dendritic growth and synaptic connectivity changes in fronto–striatal–cerebellar circuits implicated in attentional and executive domains [273]. Mechanistic reviews of PCB neurotoxicity further support a central role of these pathways, including disruption of dopaminergic neurotransmission, oxidative stress, and thyroid hormone perturbation, all consistent with an increased risk of ADHD-like alterations [74].

Overall, epidemiological studies suggest that associations between PCB exposure and ADHD-like phenotypes are present but of variable magnitude. In New Bedford, higher PCB concentrations in cord serum are associated with increased ADHD-like behaviours on Conners’ scales, particularly DSM-IV indices and the ADHD index [265,267], whereas in Duisburg prenatal exposure is more specifically associated with divided attention deficits measured by the KiTAP battery, with weaker or heterogeneous associations for parent-rated ADHD symptoms [266]. In the Mohawk sample, relationships between PCB body burden and ADHD-related outcomes appear complex and potentially modified by nutritional history and co-exposures [268]. In contrast, a pooled analysis of seven European birth cohorts did not detect a robust association between early-life PCB-153 levels and clinically diagnosed ADHD or clinically relevant symptom scores, suggesting modest effects possibly restricted to subclinical neuropsychological domains or susceptible subgroups [274]. Narrative syntheses additionally emphasise that PCB exposure is consistently linked to deficits in sustained attention, working memory, response inhibition, and cognitive flexibility—domains typically impaired in ADHD—supporting the view that PCBs may contribute to variability in ADHD-like phenotypes rather than solely categorical diagnoses [275]. Table 7 and Table 8 summarise, respectively, the main epidemiological evidence and key preclinical mechanistic findings linking PCB exposure to ADHD or ADHD-like outcomes.

## 9. Conclusions

**Figure 4:**
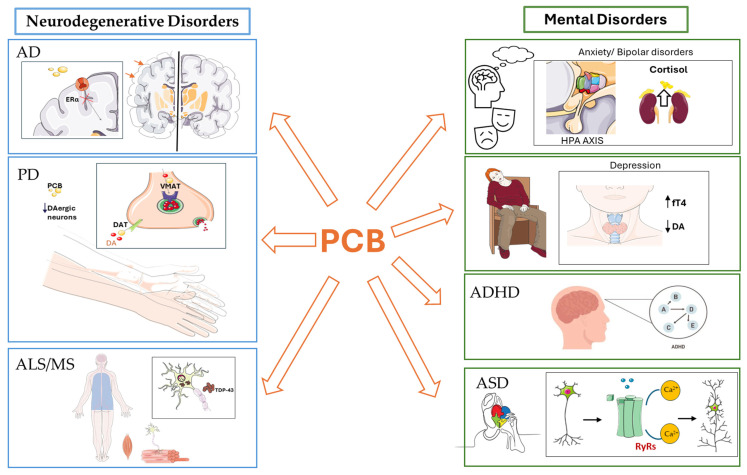
**Effects and mechanisms of PCBs on neurodegenerative, neurodevelopment and mental disorders.** This figure provides an overview of the major neurodegenerative and mental disorders that have been associated with PCB exposure, highlighting putative mechanistic links involving dopaminergic dysfunction, HPA axis alterations, and thyroid hormone imbalance in conditions such as PD, AD, ALS, depression, anxiety/bipolar disorders, and ASD. The image was created using the image bank of Servier Medical Art (Available online: http://smart.servier.com/, accessed on 20 December 2025), licensed under a Creative Commons Attribution 4.0 (CC BY 4.0) (Available online: https://creativecommons.org/licenses/by/4.0/, accessed on 20 December 2025). AD: Alzheimer’s Disease; ADHD: attention-deficit/hyperactivity disorder; ALS: Amyotrophic Lateral Sclerosis; ASD: Autism Spectrum Disorder; DA: Dopamine; HPA axis: Hypothalamic–Pituitary–Adrenal axis; PD: Parkinson’s Disease; PCBs; Polychlorinated biphenyls; T4: Thyroxine.

Accumulating evidence suggests that PCBs represent an important environmental risk factor in the onset and progression of several neurodegenerative and psychiatric disorders. In line with this, the schematic representation in Figure 4 summarizes the main neurodegenerative and mental disorders associated with PCB exposure and illustrates the putative mechanistic pathways potentially linking these conditions. The molecular pathways linking PCB exposure to neuronal injury highlight the need for integrative studies that combine epidemiology, toxicology, and molecular neuroscience. Strengthening biomonitoring efforts and developing interventions to reduce human exposure are essential steps toward preventing PCB-associated neurodegenerative outcomes.

## Figures and Tables

**Table 1:** In vitro studies showing an association between PCBs and neurodegenerative diseases.

PCB	Cell Line	PCB Treatment and Effects	Neurodegenerative Disease	Ref.
Aroclor 1254	Primary murine cortical astrocytes (C57BL6J mice)	Aroclor 1254 (1.25–50 µM, 24 h): increased oxidative stress; upregulation of antioxidant and astrocytic genes (Prdx1, Gsta2, Gfap); metabolic alterations including increased glucose uptake and ATP production (main effects at 10 µM)	PD	[85]
Aroclor 1254	Differentiated SN56 cells (murine cholinergic neuronal cell line; co-treated with Aβ25–35)	Aroclor 1254 (1, 10, 50 µM, 24 h): abolishes estradiol-mediated neuroprotection; functional anti-estrogenic effect; interferes with estrogen signaling; prevents estrogen-dependent inhibition of pathological tau phosphorylation and JNK activation	AD	[135]
Dioxin-like PCBs	BEM17 human neuroblastoma cells	Dioxin-like PCBs/AHR agonists: ~3-fold increase in TDP-43 protein; chronic exposure causes accumulation of soluble and insoluble TDP-43	ALS	[51]
Dioxin-like PCBs/AHR agonists	iPSC-derived motor neurons from ALS patients	Dioxin-like PCBs/AHR agonists (0.1 µM, 10 days): accumulation of total and insoluble TDP-43, including 35 kDa cleavage fragments	ALS	[51]

AD: Alzheimer’s Disease; ATP: Adenosine Triphosphate; Aβ: Amyloid Beta; Gfap: Glial Fibrillary Acidic Protein; Gsta2: Glutathione S-Transferase Alpha 2; JNK: c-Jun N-terminal Kinase; PD: Parkinson’s Disease; Prdx1: Peroxiredoxin 1.

**Table 2:** Experimental animal studies showing an association between PCBs and neurodegenerative diseases.

PCB	Population/Model	PCB Treatment and Effects	Neurodegenerative Disease	Ref.
Aroclor 1254	Adult male C57BL/6 mice	Aroclor 1254 (6, 12, or 25 mg/kg b.w., oral, 4 weeks): brain accumulation; dopaminergic neuron degeneration; hyperactivity; oxidative stress marker alterations; dysregulation of transferrin receptors and ferritin	PD	[84]
Aroclor 1254 + 1260	Male C57BL/6J mice (8 weeks old)	Aroclor 1254 + 1260 (7.5 or 15 mg/kg/day, 1:1 mixture): no change in striatal dopamine or tyrosine hydroxylase levels; dose-dependent decrease in DAT	PD	[59]
Mixture of coplanar (77, 126, 169) and non-coplanar PCBs (105, 118, 138, 153, 180)	AhrbCyp1a2(−/−) and AhrdCyp1a2(−/−) C57BL/6J mice (developmental exposure)	Mixture of coplanar (77, 126, 169) and non-coplanar PCBs (105, 118, 138, 153, 180): reduced spleen/thymus weights at P14; transient hypothyroxinemia; cerebellar foliation defects; altered AHR-dependent gene expression; disrupted thyroid hormone signaling; minimal evidence for PD risk	Developmental neurotoxicity	[108]
Dioxin-like PCBs/AHR agonist analogs	C57BL/6J mice	Dioxin-like PCBs/AHR agonist analogs (i.p.): ~2-fold increase in cortical TDP-43 levels	ALS	[51]

AHR: Aryl Hydrocarbon Receptor; ALS: Amyotrophic Lateral Sclerosis; b.w.: Body Weight; DAT: Dopamine Transporter; i.p.: Intraperitoneal; iPSC: Induced Pluripotent Stem Cell; PD: Parkinson’s Disease; SNpc: Substantia Nigra Pars Compacta; SOD: Superoxide Dismutase; TDP-43: TAR DNA-Binding Protein 43; TfR: Transferrin Receptor; TH: Tyrosine Hydroxylase; VTA: Ventral Tegmental Area.

**Table 3:** Epidemiological studies summarizing the association between PBSs and neurodegenerative diseases.

PCB	Population/Model	PCB Effects	Neurodegenerative Disease	Ref.
Multiple PCB congeners (including DL-PCBs)	Nested case–control study; 101 PD cases, 349 controls	Association between PCB exposure and PD development	PD	[34]
Occupational PCB exposure	Retrospective mortality study; 17,321 PCB-exposed workers	Occupational PCB exposure increased PD mortality observed in women	PD	[143]
PCB 138, 153, 180	Case–control post-mortem study; 45 PD, 14 AD, 13 controls	PCB 138, 153, 180: higher PCB levels detected in post-mortem PD brains compared to AD and controls	PD	[33]
Occupational PCB exposure	Retrospective cohort (mortality) study; 24,865 workers	Occupational PCB exposure: increased ALS mortality; no significant association with AD or PD	ALS	[127]
-	Cross-sectional cohort study; 2023 individuals (399 AD cases)	Plasma PCB levels: no association with dementia or AD prevalence	AD, dementia	[136]
PCB 118, 153, 156, 163	Prospective cohort study; 669 adults ≥65 years	PCB 118, 153, 156, 163: no association with incident dementia or AD; higher PCB levels associated with poorer cognitive performance	AD, dementia, cognitive decline	[137]
Dietary PCB exposure (seafood)	Case–control study; 79 PD cases, 154 controls	Dietary PCB exposure shows positive association with PD risk	PD	[148]
Multiple PCB congeners	Post-mortem case–control study; 45 PD, 14 AD, 13 controls	Elevated brain PCB levels associated with PD; sex-specific increases observed in female PD patients	PD, AD	[33]
PCB-153	Cross-sectional cohort; 174 exposed individuals	Altered dopaminergic metabolism, mitochondrial dysfunction, and oxidative stress pathways	PD-related neurodegenerative processes	[147]
PCB 153, 170, 180 (non-coplanar)	Cross-sectional transcriptomic study; 594 subjects	Sex-specific dysregulation of PD-related gene expression, particularly in females	PD	[149]
Wide spectrum of PCB congeners	Prospective cohort study; 699 adults >50 years	Wide spectrum of PCB congeners increased neurodegeneration risk; no clear association with PD	Dementia, PD	[151]
PCB 118, 138, 151, 175	Prospective cohort/survival analysis; 167 ALS patients	Higher PCB exposure associated with increased mortality	ALS	[154]
Chronic PCB exposure	Prospective longitudinal cohort; Faroese population (28 ALS cases)	Chronic PCB exposure did not increase ALS incidence	ALS	[158]
PCB 151, 202	Case–control study; 156 ALS cases, 128 controls	PCB 151, 202 increased ALS risk; potential mechanisms include excitotoxicity and calcium dysregulation	ALS	[156]
PCB 28, 52, 153	Case–control study; 38 ALS cases, 38 controls	PCB 28, 52, 153 weak and unstable association with ALS risk, limited to older males	ALS	[159]
PCB 28, 175, 202	Retrospective cohort study; ~26,000 ALS cases	PCB 28, 175, 202 increased ALS risk in a large U.S. cohort	ALS	[160]
Multiple PCB congeners	Cross-sectional biomarker study; 164 ALS patients, 105 controls	Multiple PCB congeners: cumulative exposure associated with increased ALS risk and poorer survival	ALS	[155]
DL-PCBs vs. NDL-PCBs	Prospective cohort study; 56,862 Finnish adults	DL-PCBs vs. NDL-PCBs: DL-PCBs showed a non-significant trend toward increased ALS risk	ALS	[161]
Hydroxylated PCB metabolites (3-OH-CB153, 4-OH-CB187	Cross-sectional case–control study; 1814 subjects	Hydroxylated PCB metabolites have a significant association with MS risk and disability progression	MS	[162]

AD: Alzheimer’s Disease; ALS: Amyotrophic Lateral Sclerosis; DL-PCBs: Dioxin-Like Polychlorinated Biphenyls; MS: Multiple Sclerosis; NDL-PCBs: Non-Dioxin-Like Polychlorinated Biphenyls; PD: Parkinson’s Disease.

**Table 4:** Studies showing an association between PCBs and mental disorders.

PCB Congener and Observed Effect	Population/Model	Neurodegenerative Disease	Ref.
PCB 126—anxiety-related behaviors	Rats	Anxiety	[172]
PCB 126—anxiety-related behaviors	Rats	Anxiety	[173]
6 NDL-PCBs—increased anxiety-like behavior after neonatal exposure	Mice	Anxiety	[174]
PCB 126, 138, 153, 180—increased activity in EPM and light–dark box	Wistar rats	Anxiety	[175]
PCB 153—higher anxiety and unhappiness	Inuit children	Anxiety	[176]
Aroclor 1254—altered corticosterone, CRH, ACTH; anxiety behaviors	Sprague–Dawley rats	Anxiety	[181]
Aroclor 1221—elevated corticosterone (females), anxiety behaviors	Sprague–Dawley rats	Anxiety	[183]
School-air PCB mixture—anxiety-like behavior, memory impairment	Female Sprague–Dawley rats	Anxiety	[185]
Aroclor 1221 + Vinclozolin—transgenerational anxiety behaviors	Female rats (F1–F6 generations)	Anxiety	[186]
PCB mixture exposure	Humans	Depression	[188]
PCB mixture exposure	Humans	Depression	[189]
PCB mixture exposure	Humans	Depression	[190]
PCB exposure—disrupted dopamine signaling, ↓ HVA	Animal/Human	Depression	[35]
PCB exposure—interference with tyrosine hydroxylase, DA synthesis	Rats	Depression	[113]
PCB exposure—inverse PCB–HVA relationship mediating depression	Adults (HELPcB study)	Depression	[202]
PCB exposure—depressive symptoms mediated via HVA	Adults (HELPcB study)	Depression	[203]
Lower-chlorinated & dioxin-like PCBs—altered thyroid hormones & HVA	Adults (HELPcB study)	Depression	[192]
PCB exposure—increased depressive symptoms over 14 years	Older adults, NY contaminated area	Depression	[193]
PCB 95—increased dendritic growth and spine density via RyRs	Rat hippocampal neuron cultures (in vitro)	Schizophrenia/Neurodevelopment	[91]
Multiple PCB congeners—no association with maternal PCB levels	Finnish birth cohort (offspring)	Schizophrenia	[200]

EPM: Elevated Plus Maze; HVA: Homovanillic Acid; RyRs: Ryanodine Receptors; DA: Dopamine; CRH: Corticotropin-Releasing Hormone; ACTH: Adrenocorticotropic Hormone; NDL-PCBs: Non-Dioxin-Like Polychlorinated Biphenyls; ↓ Decreased levels.

**Table 5:** Association of Prenatal PCB Exposure (Single Congeners and Mixtures) with Neurodevelopmental Disorders: A Synthesis of Human Cohort Studies and Meta-Analyses.

PCB Congeners	Epidemiological Study Type	Population	Effect on Brain	Brain Disease	Ref.
Generic (Principal Components–PCs)	Longitudinal Cohort Study	Mothers and Children (Prenatal exposure)	Impact on early cognitive development. Association with the emergence of autistic behaviors.	ASD	[225]
Nonspecific (Endocrine-Disrupting Chemicals–EDCs)	Systematic Review (Including 8 PCB studies)	Various Epidemiological Cohorts	No Consistent Association found between prenatal exposure and autistic traits. Highlights methodological heterogeneity.	ASD	[251]
Generic (Chemical Mixtures)	Cohort Study (Pregnancy)	Mothers and Fetuses/Children	Alteration of maternal biomarkers (e.g., decreased Vitamin D). Indicates an impact on maternal metabolic health.	Neurodevelopment	[219]
Generic (Measured in Serum/Cord Blood)	Cohort Study	Mothers and Fetuses/Children (Prenatal exposure)	Reduced birth weight and fetal head circumference.	Neurodevelopment	[227]
PCB 138	Systematic Review & Meta-Analysis	Global Birth and Population Cohorts	Increased risk of onset.	ASD	[247]
Mixtures (4 congeners	Epidemiological Study (Cohort–MIREC, Canada)	Mothers and Children (*n* = 601, 3–4 years old)	Stronger association with autistic behaviors (SRS-2 T-scores) in participants with low folic acid supplementation. PCBs may interact with maternal folate status.	ASD-like	[263]
PCB-153, PCB-180	Case–Control	Jamaican Children (2–8 years old) (169 pairs)	Inverse Association: lower odds of having serum concentrations of PCB-153 and PCB-180. Lower geometric mean concentration of 4,4′-DDE in ASD cases.	ASD	[238]
Sum of 4 PCBs (PCB118, 138, 153, 180)	Prospective Cohort (MIREC)	Canadian Mothers (first trimester samples) and children (478 pairs)	Higher gestational levels of PCBs and metals (lead, cadmium) associated with mild increases in SRS scores	Autistic Behaviors (SRS scores)	[243]
11 PCBs (e.g., PCBs 28, 74, 99, 118, 138/158, 153, 170, 180, 187, 196/203)	Cohort (EARLI) (Mixture study)	US Mothers (prenatal serum samples) and children (154 participants)	Independent positive effects on MSEL-ELC (cognitive) and VABS (adaptive) for PCB 180 and PCB 187. Higher PBDEs (47, 99) associated with greater social deficits (higher SRS scores). No overall mixture effect observed.	ASD-related traits (SRS, MSEL-ELC, VABS scores)	[242]
PCB 138/158, PCB 153, PCB 170, PCB 180	Case–Control (E MA)	US Mothers (mid-pregnancy serum samples)	Associated with increased risk of ASD. The Finnish pilot study observed an OR = 1.91 for autism for total PCBs at or above the 90th percentile.	ASD and Intellectual Disability	[233]
PCB 153 + 168, PCB 170, PCB 180 + 193, PCB 187	Prospective Cohort (MARBLES) (WGCNA analysis)	US Mothers (delivery serum) and placenta (147 samples)	Placental DNA co-methylation modules correlated with maternal serum PCB levels and child neurodevelopmental outcomes	ASD and Neurodevelopment (Mullen, ADOS scores)	[250]
PCB 118, PCB 138, PCB 153, PCB 170, PCB 180	Meta-Analysis	12 studies of cohort and case–control (4946 participants)	Significant increase in aggregated risk of ASD for exposure during pregnancy. PCB138 showed a significant correlation	ASD	[236]
PCB 118, PCB 180	Analytical Method (GC-MS/MS)	Adults and children (ASD probands and neurotypical controls) in the US (183 subjects)	Detected at high frequencies in plasma (>85% of samples). Adults had higher levels of most PCBs than children.	ASD (Cohort used for method demonstration)	[249]
Beta-Hexachlorocyclohexane (β−HCH)	Prospective Birth Cohort (HUMIS)	Norwegian children (1199 pairs)	Increased risk of ASD in children exposed to the highest quartile of β−HCH in breast milk.	ASD	[239]
Dioxins (TCDD/TEQ)	Cohort Follow-up	Vietnamese children (Bien Hoa cohort)	High TCDD exposure decreased the percentage of Face Fixation Duration in boys and lowered the percentage of Eye Fixation Duration in girls when viewing conversation scenes at 2 years old. TCDD exposure increased autistic traits (ASRS scores) in both sexes at 3 years old.	Increased Autistic Traits (ASRS)	[244]
PCBs (General), Organochlorines, Organophosphates	Systematic Review	Six case–control studies	High rates of association found between early exposure to agricultural pesticides (organochlorines, OPs, carbamates, pyrethroids) and autism. Divergences were found regarding the role of PCBs.	ASD	[237]

ASD: Autism Spectrum Disorder; DDE: Dichlorodiphenyldichloroethylene (4,4′-DDE); EDC: Endocrine-Disrupting Chemicals; EARLI: Early Autism Risk Longitudinal Investigation; GC-MS/MS: Gas Chromatography–Tandem Mass Spectrometry; HUMIS: Human Milk Study (Norwegian prospective birth cohort); MIREC: Maternal–Infant Research on Environmental Chemicals; MSEL-ELC: Mullen Scales of Early Learning—Early Learning Composite; PBDE: Polybrominated Diphenyl Ether; PCB: Polychlorinated Biphenyl; PCs: Principal Components; SRS/SRS-2: Social Responsiveness Scale (version 2); TEQ: Toxic Equivalents; TCDD: 2,3,7,8-Tetrachlorodibenzo-p-dioxin; VABS: Vineland Adaptive Behavior Scales; WGCNA: Weighted Gene Co-expression Network Analysis.

**Table 6:** Impact of PCB Mixtures on Neuronal Gene Pathways: Mechanistic Evidence from In Vivo and in vitro Models of Neurodevelopmental Disorders.

PCB Congeners	Experimental Study Type	Population	Effect on Brain	Brain Disease	Ref.
Mixtures (MARBLES Mix)	In Vivo Study (Mouse Model)	Female mice (Wild Type and Mecp2e1/Rett Syndrome mutant)	Dysregulation of 71 metabolic/gene pathways shared with MeCP2 mutation. Suggests a molecular intersection between PCB exposure and Rett Syndrome etiology.	Rett Syndrome and Neurodevelopmental Disorders	[264]
PCB138	In Vitro	Mixed neuronal/glial cultures derived from hiPSC	Chemicals working through similar or dissimilar MoA (including PCB138 in the dissimilar group) induce DNT effects in mixtures, characterized by impaired synaptogenesis (the most sensitive endpoint), neurite outgrowth, and altered BDNF levels	Learning and Memory Impairment	[253]
Mixture of 12 PCB congeners	In Vivo (Murine Model)	Mice exposed prenatally (GD18 fetuses/placenta)	Alters the DNA methylome in the placenta and fetal brain. Differentially Methylated Regions overlap significantly and are enriched for Wnt signaling and Slit/Robo signaling pathways, which are relevant to Neurodevelopmental Disorders and ASD	Neurodevelopmental Disorders, ASD	[254]
Beta-Hexachlorocyclohexane (β−HCH)	In Vivo (Zebrafish Larvae)	Zebrafish embryos and larvae (Danio rerio)	Exposure to β−HCH leads to increased proliferative cells in the optic tectum and altered social behavior (reduced shoaling/increased inter-individual distance), suggestive of neurotoxicity. Rescue experiments suggested disruption of dopaminergic neurons as a potential underlying mechanism	ASD	[239]

ASD: Autism Spectrum Disorder; BDNF: Brain-Derived Neurotrophic Factor; DNT: Developmental Neurotoxicity; GD: Gestational Day; hiPSC: Human Induced Pluripotent Stem Cell; MeCP2: Methyl-CpG-Binding Protein 2; MoA: Mode of Action.

**Table 7:** Epidemiological and review studies evaluating PCB exposure and ADHD or ADHD-like outcomes.

PCB Congeners	Epidemiological Study Type	Population and Exposure Window	PCB Exposure Assessment	Association with ADHD	ADHD/Attention Assessment Type	Ref.
Mixture of PCB congeners	Prospective birth cohort (epidemiological clinical study)	New Bedford cohort, 607 children assessed at 7–11 years; prenatal exposure in a community near a PCB-contaminated harbour	Cord serum concentrations of multiple PCB congeners (including PCB-153)	Higher prenatal PCB concentrations were associated with increased ADHD-like behaviours, particularly higher scores on DSM-IV hyperactive–impulsive symptoms and the ADHD index, especially in the highest exposure categories.	ADHD-related behaviours rated with Conners’ Rating Scales-Teacher, DSM-IV symptom scales and ADHD index	[265]
PCB-153	Prospective birth cohort with PBPK modelling (epidemiological clinical study)	441 children from the New Bedford cohort; prenatal and postnatal exposure during the first year of life	Measured cord blood PCB-153 and modelled monthly PCB-153 levels from birth to 12 months using a physiologically based pharmacokinetic model	Cord serum PCB-153 levels at birth were associated with higher ADHD-related behaviour scores, whereas associations with modelled postnatal PCB-153 exposure were weaker, suggesting a greater impact of prenatal exposure.	ADHD-related behaviours at age 8 years assessed with CRS-T indices	[267]
Multiple PCBs	Prospective birth cohort (epidemiological clinical study)	Duisburg birth cohort, 117 children; prenatal and early postnatal exposure	PCB and PCDD/F concentrations in maternal blood and breast milk	Prenatal PCB exposure was associated with more omission errors in divided attention tasks, indicating subtle attention deficits; associations with parent-rated ADHD symptoms were weak or inverse, highlighting heterogeneity of behavioural endpoints.	Attention performance assessed with KiTAP and ADHD symptoms with the FBB-ADHS parental questionnaire	[266]
PCBs congeners (118, 138 [+163 + 164], 153, 180, 74, 99, 187, 105)	Cross-sectional observational study (epidemiological clinical study)	Mohawk adolescents chronically exposed to PCBs via traditional diet; mainly childhood/adolescent exposure	Serum PCB concentrations (sum of congeners)	PCB exposure showed complex relationships with ADHD-like symptoms and cognitive outcomes, with some indications of altered attention/behaviour modulated by breastfeeding history and other co-exposures, making causal interpretation difficult.	ADHD symptoms and neuropsychological performance	[268]
PCB-153, p-p’-DDE and HCB	Pooled analysis of seven prospective birth cohorts (epidemiological clinical multi-cohort study)	Children from seven European birth cohorts; prenatal and early-life exposure up to 24 months	PCB-153 measured in maternal or cord blood and in early childhood (up to 24 months), harmonised across cohorts	No overall association was found between early-life PCB-153 exposure and clinically diagnosed ADHD or ADHD symptom scores, suggesting that if an effect exists it is small and possibly restricted to specific subgroups or co-exposure patterns.	Clinically diagnosed ADHD and ADHD symptom scales in childhood	[274]
Multiple PCBs	Narrative review (human and animal data synthesis)	Review of epidemiological and experimental studies on PCBs and lead in relation to ADHD and executive function	Not applicable (literature review)	The review concludes that PCB exposure is consistently associated with deficits in attention, response inhibition, working memory and cognitive flexibility, domains typically impaired in ADHD, supporting a role for PCBs as environmental risk factors for ADHD-like phenotypes.	ADHD diagnosis and ADHD-like neuropsychological profiles (inattention, impulsivity, executive dysfunction)	[275]

ADHD: attention-deficit/hyperactivity disorder; PCBs: Polychlorinated biphenyls.

**Table 8:** In vivo and in vitro evidence linking PCB exposure to ADHD-like neurobehavioral and mechanistic outcomes.

PCB Congeners	Experimental Study Type	Population and Exposure Window	Association with ADHD	Evaluated Endpoints	Ref.
Commercial PCB mixtures (e.g., Aroclor 1254) or defined congeners	In vivo (developmental neurotoxicity study)	Rat gestational and lactational exposure	Perinatal PCB exposure induced long-lasting alterations in locomotor activity and learning, consistent with hyperactivity and cognitive deficits in offspring, supporting an ADHD-like profile at the behavioural level.	Locomotor activity, learning and memory tasks, developmental milestones	[269]
PCB-153	In vivo	Spontaneously hypertensive rats (SHR/NCrl), and in Wistar Kyoto (WKY/NHsd) controls. Rats, postnatal dosing during early life	Postnatal PCB-153 exposure increased activity and response rates, indicating behavioural hyperactivity and impulsivity compatible with ADHD-like phenotypes.	Spontaneous activity, operant behaviour (lever pressing)	[270]
Multiple PCB congeners and mixtures	Mechanistic review (in vitro and in vivo data)	Prenatal and adult exposures	The review summarizes converging evidence that NDL-PCBs disrupt dopaminergic neurotransmission, increase oxidative stress and alter synaptic morphology, mechanisms consistent with increased risk for ADHD-like neurobehavioral alterations.	Oxidative stress, dopaminergic dysfunction, thyroid hormone disruption, synaptic plasticity	[74]

ADHD: attention-deficit/hyperactivity disorder; NDL: non-dioxin-like; PCBs: Polychlorinated biphenyls.

## Data Availability

No new data were created or analyzed in this study. Data sharing is not applicable to this article.

## Abbreviations

The following abbreviations are used in this manuscript:

ACTH	adrenocorticotropic hormone
AD	Alzheimer’s disease
ADHD	attention-deficit/hyperactivity disorder
ALS	amyotrophic lateral sclerosis
AhR	aryl hydrocarbon receptor
AhRb	high-affinity AhR
ASD	autism spectrum disorder
BFRs	brominated flame retardants
BKRM	Bayesian kernel regression models
CHARGE	(Childhood Autism Risks from Genetics and the Environment)
CNS	central nervous system
CRH	corticotropin-releasing hormone
CSHA	Canadian Study of Health and Aging
DA	dopamine
DAT	dopamine transporter
DL	dioxin-like
E2	17β-estradiol
EARLI	Early Autism Risk Longitudinal Investigation
EMA	Early Markers of Autism
ER	endoplasmic reticulum
ERS	Environmental Risk Score
FIPS-S	cohort Finnish birth-cohort design
fT4	free thyroxine
GC-MS/MS	Triple Quadrupole Gas Chromatography-Mass Spectrometry
HC-PCBs	high-chlorinated PCBs
HELPcB	Health Effects of High-Level Exposure to PCB surveillance program
HPA	hypothalamic–pituitary–adrenal
HVA	homovanillic acid
IQ	intelligence quotient
JNK	c-Jun N-terminal kinase
LC-PCBs	low-chlorinated PCBs
MIREC	Maternal-Infant Research on Environmental Chemicals
MS	multiple sclerosis
MSEL-ELC	Early Learning Composite
NDL	non-dioxin-like
O3	ozone
OCPs	organochlorine pesticides
OH-PCBs	hydroxylated PCBs
PAHs	olycyclic aromatic hydrocarbons
PCBs	Polychlorinated biphenyls
PD	Parkinson’s disease
PM	particulate matter
POPs	Persistent Organic Pollutants
RNS	reactive nitrogen species
ROS	reactive oxygen species
RyRs	ryanodine receptors
SHR	Spontaneously Hypertensive Rats
SLD	specific learning disorders
SNpc	substantia nigra pars compacta
SRS	Social Responsiveness Scale
T3	triiodothyronine
T4	thyroxine
TCDD	2,3,7,8-tetrachlorodibenzo-p-dioxin
TDP-43	Transactive response DNA-binding protein 43 kDa
TH	Thyroid hormones
THR	thyroid hormone receptor
VABS	Vineland Adaptive Behavior Scales
VMAT2	vesicular monoamine transporter 2
VOCs	volatile organic compounds
VTA	ventral tegmental area
WGCNA	Weighted Gene Correlation Network Analysis
